# Turning off inflammation naturally via dual antioxidant and anti-inflammatory actions of chestnut wood extract through PPARγ and NF-κB pathways

**DOI:** 10.1371/journal.pone.0347987

**Published:** 2026-04-29

**Authors:** Dylan Lambert, Mathys Buisine, Muriel Billamboz, Samir Jawhara

**Affiliations:** 1 CNRS, UGSF—Unité de Glycobiologie Structurale et Fonctionnelle, INSERM, France; 2 Medicine Faculty, University of Lille, Lille, France; 3 CHU Lille, Service de Parasitologie Mycologie, Pôle de Biologie Pathologie Génétique, Lille, France; 4 ICL, JUNIA, Université Catholique de Lille, LITL, Lille, France; Emory University School of Medicine, UNITED STATES OF AMERICA

## Abstract

**Background:**

Inflammatory bowel disease (IBD) arises from a persistent imbalance between oxidative stress and immune homeostasis, driving tissue injury and chronic intestinal inflammation. Natural polyphenols are increasingly recognized as powerful modulators of redox and immune pathways, yet the bioactivity of complex, tannin-rich extracts remains largely overlooked. Among these, chestnut wood extract (CWE) particularly rich in highly soluble tannins, represents a valuable yet underexplored reservoir of bioactive molecules. This study aimed to characterize the phytochemical diversity of CWE and evaluate its anti-inflammatory, antioxidant, and epithelial-protective properties.

**Methods and Results:**

Through a comparative screening of polyphenol-enriched plant extracts, chestnut wood extract (CWE) emerged as a standout candidate displaying potent anti-inflammatory and antioxidant effects. Phytochemical profiling revealed 23 distinct phenolic constituents, including phenolic acids, gallotannins, ellagitannins (castalagin, vescalagin), roburins, and ellagic diglucosides, highlighting its high chemical diversity. Functionally, CWE strongly attenuated NF-κB activation and reduced pro-inflammatory cytokines (IL-6, IL-1β, TNF-α) in human macrophages and Caco-2 intestinal epithelial cells. It also mitigated oxidative stress by reducing intracellular ROS and upregulating key antioxidant enzymes (SOD-1, GPX-1, catalase), while restoring mitochondrial membrane potential. Mechanistically, CWE enhanced PPARγ expression and behaved as a selective PPARγ modulator, synergizing with rosiglitazone and controlling IL-6 via both PPARγ-dependent and -independent pathways. Remarkably, CWE preserved intestinal epithelial barrier integrity and boosted Claudin-1 expression under inflammatory challenge. In vivo, CWE improved *Caenorhabditis elegans* survival following *Candida albicans* infection, supporting its protective capacity against oxidative and pathogenic challenges.

**Conclusion:**

Collectively, these findings unveil CWE as a chemically rich, biologically active plant extract with dual antioxidant and anti-inflammatory properties. By restoring redox balance, modulating PPARγ signaling, and preserving epithelial integrity, CWE represents a promising natural candidate for mitigating intestinal inflammation.

## Introduction

Chronic inflammatory bowel diseases (IBD), including Crohn’s disease and ulcerative colitis, are relapsing disorders characterized by recurrent mucosal inflammation and impaired intestinal homeostasis. Conventional therapies ranging from dietary management to pharmacological and surgical interventions, aim to control inflammation and sustain remission [1–4]. However, these approaches remain palliative, long-term administration of corticosteroids, immunomodulators, or anti-TNF-α antibodies often leads to adverse effects, tolerance, or therapeutic resistance, eventually necessitating surgery in severe cases [4–7]. This clinical reality underscores the urgent need for safer, more sustainable therapeutic strategies [8,9].

Among emerging candidates, plant-derived polyphenols have gained considerable attention due to their pleiotropic biological activities [10]. These secondary metabolites exhibit antioxidant, anti-inflammatory, and immunomodulatory properties that contribute to both systemic and gastrointestinal health [11]. Beyond their direct effects, polyphenols interact with the gut microbiota, which metabolizes them into bioactive compounds, such as urolithin B that further enhance intestinal and metabolic resilience [5,12,13]. Although polyphenols are widely described as beneficial bioactive compounds, their biological effects are highly dose-dependent. At high concentrations some polyphenols may exhibit pro-oxidant properties [14,15]. Furthermore, their generally low intestinal absorption and extensive metabolism limit systemic exposure to intact compounds, complicating direct extrapolation from in vitro findings to in vivo settings.

Specific polyphenols, including tannins (ellagic and gallic acids) and flavonoids (quercetin, apigenin, curcumin), have demonstrated efficacy in maintaining intestinal integrity by modulating inflammatory pathways (NF-κB, MAPK), enhancing epithelial barrier function, and restoring microbial balance [16,17]. Notably, quercetin has been shown to reinforce antioxidant defenses by upregulating GPX-1, SOD-1, and catalase, while preserving mucin production and tight junction structure [18, 19].

Recent advances also highlight the potential of polyphenol-rich extracts obtained from agricultural and forestry by-products, such as grape seeds, pomegranate, tea, quebracho (*Schinopsis spp*.), and chestnut (*Castanea sativa Mill*.) which are often discarded despite their remarkable bioactive potential [11,20]. These extracts have already been successfully used as natural alternatives to antibiotics in animal nutrition, improving intestinal health and immune function [13,21,22].

Due to their high polyphenolic content, metabolic versatility, and multifaceted biological effects, such natural extracts represent promising candidates for the prevention and management of chronic intestinal inflammation [22–25]. Harnessing these bioresources could pave the way toward innovative, sustainable, and microbiota-friendly therapeutic strategies for IBD [26–29].

In light of this, the present study investigates the potential of various plant-derived extracts to modulate the inflammatory response, with a particular focus on the anti-inflammatory and antioxidant activities of chestnut wood extract (CWE) from *Castanea sativa Mill*. To bridge the gap between biochemical effects and physiological relevance, a *Caenorhabditis elegans* infection model was employed, using *Candida albicans* to mimic in vivo conditions of infection and inflammation. This integrative approach allowed us to evaluate how CWE influences immune response modulation during infection and to determine whether CWE treatment enhances the overall health and resilience of the nematode host. In this study, we aim to provide novel insights into the immunomodulatory potential of plant extracts and their promising applicability in managing inflammation-related disorders.

## Materials and methods

### Human and fungal cell culture

The human monocytic cell line THP-1 (InvivoGen, France) is cultured at 37°C in RPMI medium supplemented with 10% fetal bovine serum (FBS) and 1% antibiotics (penicillin and streptomycin). These cells are incubated at 37°C with an atmosphere containing 5% CO_2_. The differentiation of THP-1 cells into macrophages is induced by treating these THP-1 cells with phorbol myristate acetate (PMA) at a concentration of 200 ng/ml for 48 hours. The human intestinal epithelial cell line Caco-2 is incubated at 37°C with 5% CO_2_ in DMEM (Dulbecco’s Modified Eagle Medium) containing 20% FBS and 1% antibiotics (penicillin and streptomycin). THP-1-NF-κB cells (InvivoGen, France) are genetically modified, transfected with an NF-κB-luciferase reporter gene [30]. These cells are cultured in RPMI medium containing 20% FBS and 1% antibiotics (normocin and zeocin). The wild-type strain of *C. albicans* SC5314 is cultured in Sabouraud liquid medium at 37°C for 24 hours.

### CWE fractionation

Lyophilized CWE (20 g) was partitioned using ethyl acetate (EtOAc) and water. The EtOAc layer was dried and lyophilized (3.1 g), and subjected to preparative HPLC to yield gallic acid (1, 21.2 mg) and ellagic acid (2, 15.4 mg) [31]. The aqueous layer was adsorbed onto XAD-7 resin, washed with water to remove polar impurities, and eluted with ethanol to obtain a polyphenol-enriched extract (14 g). This extract was fractionated into eight major fractions (FI–FVIII) by centrifugal partition chromatography (CPC) using various biphasic solvent systems (ascending and dual modes). Further purification by Sephadex LH-20 and preparative HPLC led to the isolation of 23 polyphenolic compounds (in Supplementary data S1 Fig) [31].

### Quantification of NF-κB Expression in Transgenic THP-1-NF-κB Cells

THP-1-NF-κB cells were incubated at a concentration of 7 x 10^4^ cells/mL in a 96-well plate [30]. They were then exposed to 80 μg/mL of various natural extracts used in this study (the list of the different extracts as well as the controls used is summarized in Table 1).

**Table 1:** List of natural extracts and polyphenolic compounds analyzed in this study.

Product name	Abbreviations	Concentration	Supplier	References
Chestnut wood extract	CWE	80 µg/mL	KingTree society, France	[31]
Light Oak Wood Extract	LOWE	80 µg/mL		[54]
Dark Oak Wood Extract	DOWE	80 µg/mL		[54]
Cherry Wood Extract	ChWE	80 µg/mL		[55]
Cashew Extract	CE	80 µg/mL	Green’ing society, France	[56]
Occitania Grape Seed Extract	OGSE	80 µg/mL		[57]
Thyme Extract	ThE	80 µg/mL		[58]
Occitania Corroyere Extract	OCE	80 µg/mL		[59]
Urolithin B	Uro B	100 µM	Sigma-Aldrich, France	[12]
Quercetin	Q	100 µM		[60]

Concentrations of plant extracts (complex mixtures) are expressed in µg/mL, while those of pure polyphenolic control compounds are expressed in µM.

CWE were used at a concentration of 20 µg/mL, 26 µg/mL, 40 µg/mL, 80 µg/mL, 160 µg/mL, 300 µg/mL and 600 µg/mL in PBS. Other naturals extracts were assessed at a concentration of 100 µg/mL in PBS and other controls polyphenols were used at a concentration of 100 µM first in DMSO and then diluted on PBS.

For urolithin B (CAS 1139-83-9) or quercetin (CAS 849061-97-8), a volume of 100 μM of urolithin or quercetin was added to cells. These THP-1-NF-κB cells were then stimulated with LPS (LPS from *E. coli* O111: B4; Sigma-Aldrich, France) at a concentration of 250 ng/mL, for 24 hours at 37°C in a 5% CO_2_ atmosphere. After 24 hours of incubation, a 20 μL aliquot of supernatant was collected and transferred to a black 96-well plate containing 50 μL of luciferin substrate (Quanti-lucTM 4 Lucia/Gaussia; Invivogen, Lot No: 10613−4504). The luciferase activity was quantified by measuring bioluminescence with a spectroluminometer to determine NF-κB expression levels.

### Quantification of mitochondrial integrity

Caco-2 cells are incubated at a concentration of 1 × 10^6^ cells/ml in a 96-well plate. Caco-2 cells were observed daily under an inverted microscope to ensure confluence and tight cell-to-cell adhesion which are indicative of monolayer integrity (confluence and structural organization of Caco-2 monolayers were confirmed as illustrated in Supplementary data S2 Fig). These intestinal cells are then exposed to 2% dextran sulfate sodium (DSS, MP Biomedicals, LLC, Eschwege, Germany) and treated with CWE at a concentration of 80 µg/mL for 24 h at 37°C and 5% CO_2_. Cell viability is measured according to the manufacturer’s protocol (JC-10 Mitochondrial Membrane Potential Assay Kit microplate, Abcam, Pays). This kit relies on the labeling of various conformations of the JC-10 mitochondrial protein. During apoptosis, the mitochondrial membrane becomes depolarized, leading to a change in the conformation of the JC-10 protein from aggregated to monomeric form. Mitochondrial depolarization is quantified spectrophotometrically at 490–525 nm for the monomeric form of JC-10 and at 540–590 nm for the aggregated form. The monomer/aggregate ratio is then calculated to assess mitochondrial integrity in intestinal cells during the inflammatory response.

### Quantification of inflammatory response mediators

Macrophages and intestinal Caco-2 cells were incubated at a density of 1 x 10^6^ cells/mL in a 24-well plate [32]. Macrophages were then treated with CWE at 80 μg/mL, or 100 μM of urolithin B or quercetin. These cells were then stimulated with LPS at a concentration of 250 ng/mL, for 24 hours at 37°C in 5% CO_2_. For intestinal Caco-2 cells, they were treated under the same conditions as above but exposed to 2% DSS (MP Biomedicals, LLC, Eschwege, Germany) instead of LPS. For PCR, total RNA was extracted by lysing macrophages and Caco-2 cells with 350 μL of RA1 lysis buffer following the NucleoSpin RNA® protocol (Macherey-Nagel). cDNA synthesis was performed using the High-Capacity cDNA Reverse Transcription Master Mix (Applied Biosystems). Gene expression levels of inflammatory mediators were quantified relative to the housekeeping gene GAPDH using Sybr Green (Fast Sybr Green Master Mix; Thermo Fisher Scientific, Applied Biosystems) on a StepOne real-time PCR system (Applied Biosystems, USA) [33,34]. For protein quantification of IL-6 and IL-1β production in macrophages, the protein levels of pro-inflammatory cytokines IL-6 and IL-1β in the supernatants of THP-1 cells treated with CWE, urolithin B, or quercetin were quantified using ELISA kits for IL-6 (Assay Genie Human IL-6 ELISA Kit, Assay Genie) and IL-1β (BD OptEIA^TM^ ELISA Kit, BD Bioscience, San Diego, CA, USA), following the manufacturers’ instructions. IL-6 and IL-1β concentrations were measured at 450 nm and 570 nm using a spectrophotometer (FLUOstar® Omega; BMG Labtech, Saitama, Japan). For quantification of ROS production in macrophages, cells were incubated at a concentration of 1 x 10^5^ cells/mL in a 96-well plate. Macrophages were then treated with 80 μg/mL of CWE, or 100 μM of urolithin B or quercetin. Macrophages were then stimulated with LPS (LPS from *E. coli* O111: B4; Sigma-Aldrich, France) at a concentration of 250 ng/mL for 3 hours at 37°C in a 5% CO_2_ atmosphere. ROS production was quantified using 50 μM luminol (Réf: 123072−2.5G, Sigma-Aldrich, France) and 5 U of HRP peroxidase (Réf: P8375-1KU, Sigma-Aldrich, France) by measuring bioluminescence with a spectroluminometer (FLUOstar® Omega; BMG Labtech, Saitama, Japan). For Western blot analysis, proteins were extracted from Caco-2 cells using 350 μL of RA1 lysis buffer according to the NucleoSpin RNA/Protein® protocol (Macherey-Nagel). Protein concentration was determined using a NanoDrop spectrophotometer (DeNovix Inc., USA), and samples were adjusted to 25 μg of total protein prior to separation by SDS-PAGE on 10% acrylamide gels. Proteins were subsequently transferred onto membranes using the iBlot™ 2 system (Invitrogen, Thermo Fisher Scientific). Membranes were incubated with the following primary antibodies: phospho-NF-κB p65 (Ser536) monoclonal antibody (1:20,000; Euromedex, France), NF-κB p65 monoclonal antibody (2 μg/mL; Bio-Techne, France), PPAR-γ (phospho-Ser273) monoclonal antibody (1:1,000; Antibodies-online, Germany), and β-actin (0.01 μg/mL; Bio-Techne, France). After washing, membranes were incubated with horseradish peroxidase (HRP)-conjugated secondary antibodies directed against mouse IgG (Sigma-Aldrich, France) or rabbit IgG (Zymed, USA). Protein bands were visualized by enhanced chemiluminescence using ECL reagents (Thermo Fisher Scientific, France) and detected with the iBright™ 1500 imaging system (Invitrogen, Thermo Fisher Scientific).

### PPARγ activation and inhibition in macrophages

Macrophages were incubated at a density of 1 × 10⁶ cells/mL in 24-well plates. Cells were treated with CWE (80 μg/mL), Bisphenol A diglycidyl ether (A, 100 μM, Sigma Aldrich, France), Rosiglitazone (RSG, 50 nM, Sigma Aldrich, France), or combinations of A + CWE (100 μM + 80 μg/mL) or RSG + CWE (50 nM + 80 μg/mL). Following treatment, macrophages were stimulated with lipopolysaccharide (LPS) at a final concentration of 250 ng/mL and incubated for 24 hours at 37 °C in a humidified atmosphere containing 5% CO₂. For gene expression analysis, total RNA was extracted using 350 μL of RA1 lysis buffer according to the NucleoSpin RNA® protocol (Macherey-Nagel). Complementary DNA (cDNA) was synthesized from the isolated RNA using the High-Capacity cDNA Reverse Transcription Master Mix (Applied Biosystems). Quantitative real-time PCR was performed using Fast SYBR™ Green Master Mix (Thermo Fisher Scientific, Applied Biosystems) on a StepOne Real-Time PCR System (Applied Biosystems, USA). Expression levels of target genes were normalized to the housekeeping gene GAPDH and analyzed using the comparative Ct method.

### Quantification of intestinal permeability

Intestinal Caco-2 cells were incubated at a concentration of 1 x 10^5^ cells/mL in transwell inserts (HTS Transwell 96-well; Corning; United Kingdom) to form an intestinal barrier. Caco-2 cells were cultured on Transwell inserts under conditions allowing full polarization and differentiation. The differentiation status was carefully monitored throughout the experiment. Caco-2 cells were observed daily under an inverted microscope to ensure confluence and tight cell-to-cell adhesion, which are indicative of monolayer integrity. The uniformity and continuity of the epithelial monolayer formed on the inserts were visually confirmed before initiating permeability assays. Caco-2 cells were then treated with 80 μg/mL of CWE, or 100 μM of urolithin B or quercetin, and 250 µg/mL of Dextran-FITC (Fluorescein isothiocyanate Dextran 70,000, Sigma-Aldrich, France) in the presence of 2% DSS (Dextran Sodium Sulfate; MP Biomedicals, LLC, Eschwege, Germany) for 24 hours at 37°C in 5% CO_2_. Intestinal barrier permeability was assessed by measuring the fluorescence emitted by Dextran-FITC that crossed the barrier at 485/520 nm using a spectrophotometer (FLUOstar® Omega; BMG Labtech, Saitama, Japan). Intestinal Caco-2 cells exposed to DSS and treated with CWE or natural extracts were fixed on IBIDI glass slides (µ-slide 8 wellhigh Glass Bottom; Ibidi cells in focus) using 10% paraformaldehyde for 10–15 minutes. The cells were permeabilized with 0.1X Triton for 10 minutes and subsequently labeled with DAPI (DNA) and PKH26 (membranes). The impact of CWE on the integrity of intestinal epithelial cells was then observed using confocal microscopy (Zeiss LSM 710 AiryScan; Bioimaging Center Lille BiCel).

### Survival of *C. elegans* Infected with *C. albicans*

The SC5314 strain of *C. albicans* was cultured in liquid Sabouraud medium at 37°C for 24 hours. Wild-type N2 strains of *C. elegans* were grown on *E. coli* OP50-seeded medium at 20 °C [35,36]. *C. elegans* were synchronized and incubated at 20°C. *C. albicans* was then plated on Brain Heart Infusion (BHI, Oxoid Ltd, United Kingdom) agar containing amikacin (45 μg/mL) for 30 minutes. Synchronized *C. elegans* were washed multiple times with M9 buffer containing 90 μg/mL amikacin to remove *E. coli* and promote ingestion of *C. albicans*. Nematodes were then incubated on BHI agar plates containing *C. albicans* for 6 hours. Following infection, nematodes were counted, and treated with 100 μg/mL of CWE in M9 buffer supplemented with 5% BHI for five days. The effects of treatments on nematode survival were assessed daily by quantifying the population of infected nematodes between treated and untreated groups. Each experiment of *C. elegans* was performed three times independently with approximately 20 nematodes per replicate

Parallel experiments were conducted to evaluate gene expression related to immune responses in nematodes. Total RNA was extracted from nematodes treated with CWE after 24 hours using the NucleoSpin RNA® kit (Macherey-Nagel, Hoerdt, France). RNA concentration was measured using spectrophotometry (Nanodrop; Nyxor Biotech, Paris, France). cDNA was synthesized using the High-Capacity cDNA Reverse Transcription Master Mix (Applied Biosystems, Foster, CA, USA). Gene expression of immune-related markers was quantified using Fast Sybr Green (Fast Sybr Green Master Mix; Thermo Fisher Scientific) in real-time PCR (Applied Biosystems). Results were normalized to the reference gene β-actin.

### Statistical analysis

Results were analyzed using GraphPad Prism® software (GraphPad Software, San Diego, CA, USA). Statistical differences between groups were determined using the Mann-Whitney test, with p-values ≤ 0.05 considered statistically significant.

## Results

### CWE’s anti-inflammatory properties

**Fig 1:**
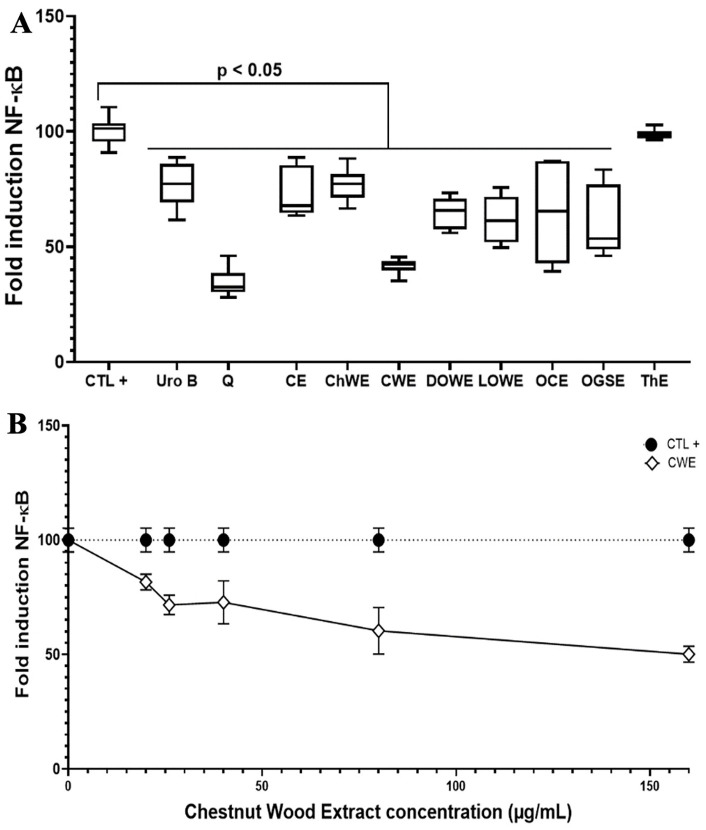
The effect of pure polyphenols and various natural extracts on NF-κB-luciferase expression in the presence of LPS and macrophages exposed to LPS alone or combined with increasing concentrations of CWE. **(A)** CTL + : macrophages were exposed to LPS; Uro B: macrophages exposed to LPS and treated with UroB; Q: macrophages exposed to LPS and treated with quercetin; CE: macrophages exposed to LPS and treated with 80 µg/mL of cashew extract (CE); ChWE: macrophages exposed to LPS and treated with 80 µg/mL of cherry wood extract (ChWE); CWE: macrophages exposed to LPS and treated with80 µg/mL of chestnut wood extract (CWE); DOWE: macrophages exposed to LPS and treated with 80 µg/mL of dark oak wood extract (DOWE); LOWE: macrophages exposed to LPS and treated with 80 µg/mL of light oak wood extract (LOWE); OCE: macrophages exposed to LPS and treated with 80 µg/mL of occitania corroyere extract (OCE); OGSE: macrophages exposed to LPS and treated with 80 µg/mL of occitania grape seed extract (OGSE); ThE: macrophages exposed to LPS and treated with 80 µg/mL of thyme extract (ThE). **(B)** CWE: macrophages exposed to LPS and treated with 20 µg/mL, 26 µg/mL, 40 µg/mL, 80 µg/mL or 160 µg/mL of CWE. Concentrations of plant extracts (complex mixtures) are expressed in µg/mL, while those of pure polyphenolic control compounds are expressed in µM. All assays were independently repeated three times with each condition tested in triplicate per experiment (n = 9), and results are presented as mean + /- standard deviation.

We evaluated the anti-inflammatory effects of plant-derived polyphenols on NF-κB, a key regulator of inflammation (Fig 1). Two well-characterized metabolites, urolithin B and quercetin, were compared with various plant extracts, revealing distinct modulatory profiles and highlighting their potential in regulating inflammatory pathways (Fig 1).

Using THP-1 macrophages stably transfected with an NF-κB-luciferase reporter, we assessed the anti-inflammatory effects of 100 µM urolithin B and quercetin. Following LPS stimulation, both compounds reduced NF-κB activity, with quercetin exhibiting a significantly stronger inhibitory effect than urolithin B (Fig 1).

Additionally, we screened several natural plant extracts, many of which are reported in the literature and by suppliers to contain high levels of polyphenols, for their anti-inflammatory effects. LPS-exposed macrophages were treated with 80 µg/mL of these extracts for 24 hours to assess their impact on NF-κB expression. Among the extracts tested, thyme extract (ThE) was the only one that showed no significant effect on NF-κB expression at this concentration. In contrast, CWE exhibited the most potent anti-inflammatory activity.

To ensure consistency and reliability in downstream experiments, the CWE batch used in this study was first subjected to detailed phytochemical characterization and compound identification (in Supplementary data S1 Table and S1 Fig). A detailed chemical characterization of CWE (Table 2) identified 23 polyphenolic compounds, belonging to the families of simple phenolic acids (e.g., gallic and ellagic acid), gallotannins, C-glucosidic ellagitannins (e.g., castalagin, vescalagin), roburins, modified ellagitannins (e.g., castacrenins), and ellagic diglucosides. This classification underscores the chemical diversity of hydrolyzable tannins in the extract. Quantification revealed that hydrolyzable tannins represent 76.7% of the total polyphenolic content, confirming their dominance within CWE. Cytotoxicity evaluation across increasing CWE concentrations, including doses up to 300 µg/mL (4 × higher than those used in mechanistic assays), revealed no adverse effects on cell viability (S2 Fig in supplementary data). Further analysis focused on CWE, revealing a dose-dependent effect on NF-κB expression in macrophages under inflammatory conditions. At concentrations as low as 20 µg/mL, CWE significantly reduced NF-κB expression in LPS-exposed macrophages. These findings highlight CWE’s potential as a powerful natural anti-inflammatory agent, emphasizing the necessity of advancing our research to better understand its underlying biological properties.

**Table 2:** Summary of the main compounds identified and characterized in the CWE.

Compound Name	Number	Molecular Formula	Structural Features	Characterization Methods	Remarks
**Castalin**	3	–	Ellagitannin	UPLC-MS, NMR	Not detected in several biphasic systems
**Castalagin**	4	–	Ellagitannin	UPLC-MS, NMR	Good retention (high KD)
**Vescalagin**	5	–	Ellagitannin	UPLC-MS, NMR	Highly polar
**Vescalagin carboxylic acid**	6	–	Carboxylated derivative of vescalagin	UPLC-MS, NMR	Present in polar fractions
**Roburin E**	7	–	Ellagitannin dimer	UPLC-MS, NMR	Very high KD
**Roburin D**	8	–	Ellagitannin dimer	UPLC-MS	Not detected in tested biphasic systems
**Castalagin A**	9	C41H30O28	Contains an NHTP (nonahydroxytriphenoyl) motif	HRMS, 2D NMR (COSY, HMBC)	Newly reported structure
**Casuariin**	10	–	Galloylated ellagitannin	UPLC-MS, NMR	Found in fractions IV and VII
**Castacrenin K**	12	–	Carboxylated derivative	HRMS, NMR	Structure confirmed by comparison with 13
**Castacrenin I**	13	–	Lacks HHDP motif	HRMS, NMR	New structure; positive optical rotation
**Castacrenin D**	14	–	m/z identical to 1-O-galloylcastalagin	Comparative LC-MS	Possible confusion with previously described compound
**Castacrenin J**	15	–	Lacks HHDP motif	NMR	New structure characterized
**Castacrenin E**	16	–	Partially hydrolyzed ellagitannin	NMR	Found in fraction VII
**Castacrenin H**	18	C22H18O14	Galloyl half + epoxy bridge (epoxyglucose)	HRMS, NMR (COSY, NOE)	New structure with stereochemistry determined
**Unnamed ellagitannins (mixture)**	19–21	–	Mixture of epimers (α/β glucose forms)	NMR, LC-MS	Difficult to separate despite HPLC purification
**1,1′;2,2′-di-HHDP-diglucose**	22	C40H36O28	Diglucose linked to two HHDP units	HRMS, 2D NMR	Revised structure, formerly named “D-6”
**4,4′;2,2′-di-HHDP-6,6′-digalloyl-diglucose**	23	C54H44O36	Galloylated diglucose with two HHDP units	HRMS, NMR (COSY, HMBC, HSQC)	Newly characterized structure

Light gray shading indicates newly identified or structurally novel compounds. HRMS, high-resolution mass spectrometry; NMR, nuclear magnetic resonance; HHDP, hexahydroxydiphenoyl; KD, distribution coefficient.

### CWE Effect on the modulation of inflammatory response in macrophage and intestinal cells

CWE’s impact on inflammatory responses was further examined by analyzing its effects on key mediators of inflammation. Additionally, we evaluated CWE’s influence on the expression of various receptors, including TLRs, in macrophages exposed to LPS and human intestinal cells exposed to 2% DSS. We chose to focus particularly on IL-6, IL-1β, and TNF-α as key pro-inflammatory cytokines since they are widely recognized as central mediators of the inflammatory response and are commonly used as reliable markers in inflammation studies. The chemokines IL-8, CCL-2, CCL-5, and CXCL-10 were also among the cytokines studied due to their important roles in recruiting immune cells and mediating inflammation.

**Fig 2:**
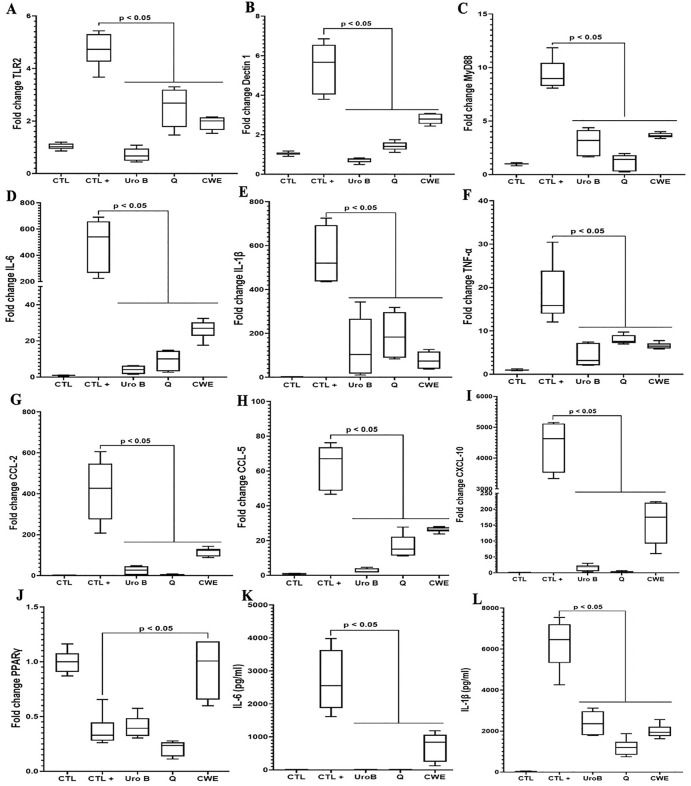
Impact of CWE treatment on the expression of immune receptors, intracellular signaling pathways, and pro-inflammatory mediators in macrophages. Expression of TLR2 **(A)**, Dectin-1 **(B)**, MyD88 **(C)**, IL-6 **(D)**, IL-1β **(E)**, TNF-α **(F)**, CCL-2 **(G)**, CCL-5 **(H)**, CXCL-10 **(I)**, and PPAR-γ (J) in LPS-stimulated and CWE-treated macrophages. **(K and L)** Analysis of IL-6 and IL-1β protein production in macrophages stimulated by LPS and treated with CWE. CTL: macrophages alone; CTL + : macrophages exposed to LPS; UroB: macrophages exposed to LPS and treated with urolithin B at a concentration of 100 µM; Q: macrophages exposed to LPS and treated with 100 µM of quercetin; CWE: macrophages exposed to LPS and treated with 80 µg/mL of CWE. CTL indicates negative controls (untreated, non-inflamed cells), whereas CTL+ refers to positive controls representing inflammatory conditions (cells exposed to pro-inflammatory stimuli). All assays were independently repeated three times with each condition tested in triplicate per experiment (n = 9), and results are presented as mean + /- standard deviation.

Similar to urolithin B and quercetin, CWE at a concentration of 80 µg/mL reduced macrophage inflammatory responses. Regarding its effect on the TLRs/NF-κB signaling pathway, CWE decreased the expression of TLR2, Dectin-1, and MyD88 (Fig 2A, 2B and 2C). These findings correlate with reduced expression of pro-inflammatory cytokines and chemokines. CWE significantly lowered the expression of pro-inflammatory cytokines, including IL-6, IL-1β, and TNF-α (Fig 2D, 2E, 2F, 2K, and 2L). Additionally, it decreased the expression of pro-inflammatory chemokines such as CCL-2, CCL-5, and CXCL-10 (Fig 2G, 2H, and 2I). We observed that PPARγ expression was restored in LPS-stimulated macrophages treated with CWE to levels comparable to those in control macrophages (Fig 2J). Similarly, to the effects observed in macrophages, CWE treatment reduced NF-κB expression in DSS-challenged Caco-2 intestinal epithelial cells, while increasing PPAR-γ expression compared with DSS-exposed, untreated cells (S3 Fig). Additionally, IL-6 protein expression was analyzed in Caco-2 cells exposed to DSS as well as in DSS-exposed cells treated with CWE. CWE treatment significantly reduced IL-6 production in intestinal cells (S3 Fig). Consistent with these results, Western blot analysis revealed increased levels of both phosphorylated and total NF-κB protein in Caco-2 cells following DSS exposure. CWE treatment markedly reduced NF-κB protein expression, which was associated with a concomitant increase in PPAR-γ protein levels in DSS-challenged cells. These findings suggest that CWE modulates the NF-κB/PPAR-γ signaling axis under inflammatory conditions (S3 Fig). To further investigate this mechanism, we assessed the effects of both a PPARγ agonist and antagonist on cytokine expression profiles associated with PPARγ signaling.

### CWE Modulates Inflammatory Responses in Macrophages via PPARγ-Dependent and Independent Mechanisms

**Fig 3:**
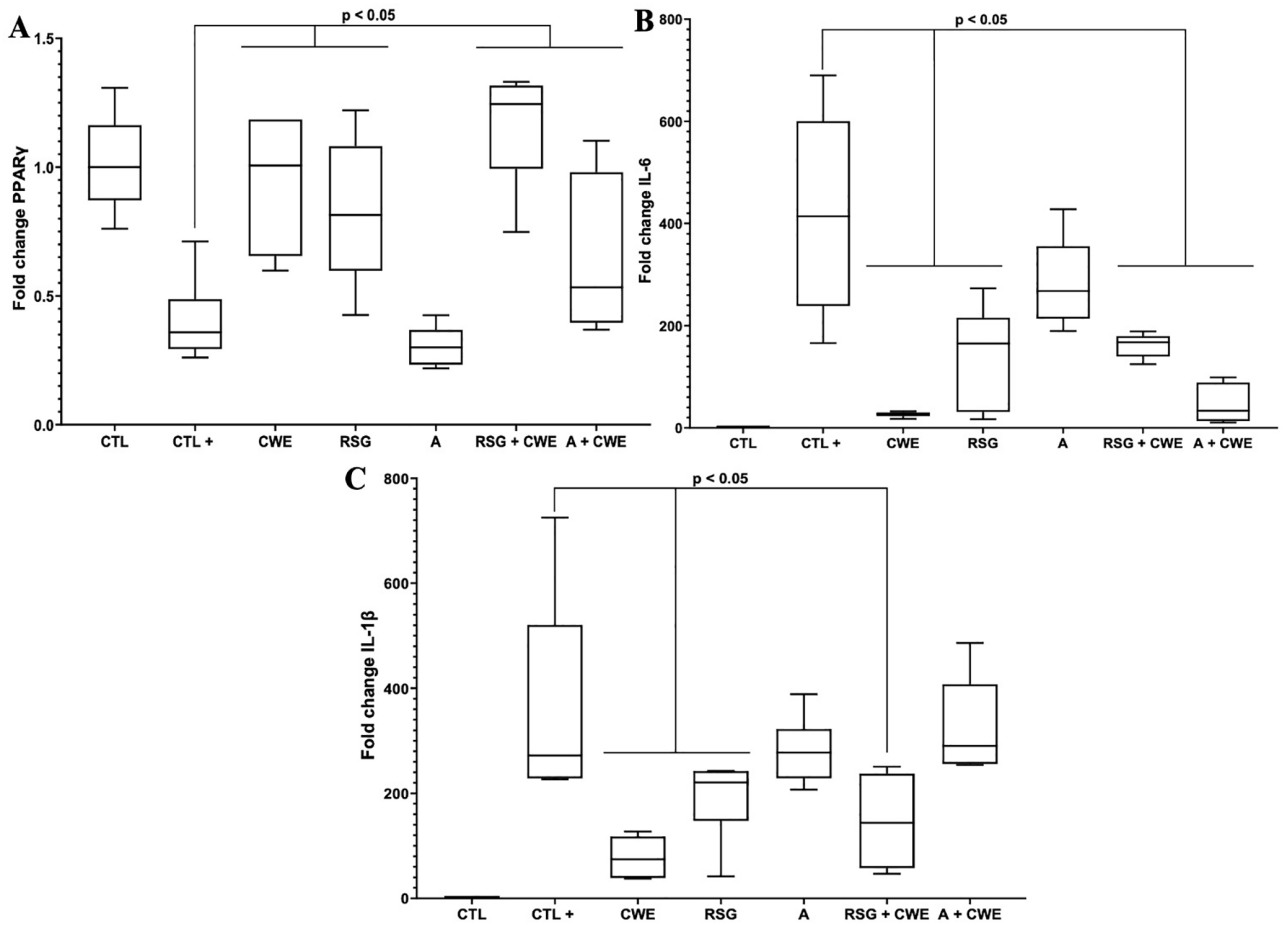
Effect of CWE treatment on the expression of PPAR-γ and pro-inflammatory mediators in macrophages. The expression of PPAR-γ **(A)**, IL-6 **(B)**, and IL-1β (C) was evaluated in macrophages stimulated with LPS and treated with CWE in combination with a PPAR-γ antagonist (A) or a PPAR-γ agonist (Rosiglitazone, RSG). Untreated macrophages served as the negative control (CTL), while LPS-stimulated macrophages without further treatment were used as the positive control (CTL+). Additional groups included macrophages treated with LPS and 100 µM of antagonist A **(A)**, or 50 nM of RSG (RSG). Another group received 80 µg/mL of CWE following LPS stimulation (CWE). To evaluate potential interactions, macrophages were also co-treated with 100 µM of PPAR-γ antagonist and 80 µg/mL of CWE (A + CWE), or with 50 nM of rosiglitazone and 80 µg/mL of CWE (RSG + CWE). All experiments were independently repeated three times, with each condition tested in triplicate per experiment (n = 9). Results are presented as mean ± standard deviation.

PPARγ agonist rosiglitazone significantly upregulates PPARγ expression in macrophages under pro-inflammatory conditions (LPS stimulation). Interestingly, treatment with CWE also enhances PPARγ expression to a comparable effect to rosiglitazone, suggesting that CWE may function as a partial or indirect agonist of PPARγ (Fig 3A). In contrast, under the same inflammatory conditions, treatment with a PPARγ antagonist alone did not increase PPARγ expression which remained at basal levels. Notably, the combined treatment of rosiglitazone and CWE resulted in an additive effect on PPARγ expression, indicating potential complementary activation mechanisms. In contrast, CWE in the presence of the PPARγ antagonist, PPARγ expression was reduced when compared to CWE or rosiglitazone alone. However, this expression remained higher than basal levels, emphasizing that CWE may still activate PPARγ. Regarding pro-inflammatory markers, both CWE and rosiglitazone alone significantly reduced the expression of IL-1β and IL-6. In contrast, PPARγ antagonist treatment maintained cytokine expression levels comparable to the positive control group (LPS-stimulated macrophages) confirming the role of PPARγ in mediating anti-inflammatory effects. Besides, co-treatment with CWE and rosiglitazone under inflammatory conditions led to a further significant reduction in IL-1β and IL-6 levels, reinforcing the notion of additive effects on the transcriptional regulation of inflammatory genes (Fig 3B and 3C). However, in the presence of the PPARγ antagonist, CWE failed to decrease IL-1β, suggesting that its anti-inflammatory effect on this cytokine is predominantly PPARγ-dependent. Intriguingly, CWE was still able to reduce IL-6 expression despite PPARγ inhibition, highlighting the involvement of alternative signaling pathways beyond PPARγ in modulating IL-6 expression via CWE (Fig 3B).

**Fig 4:**
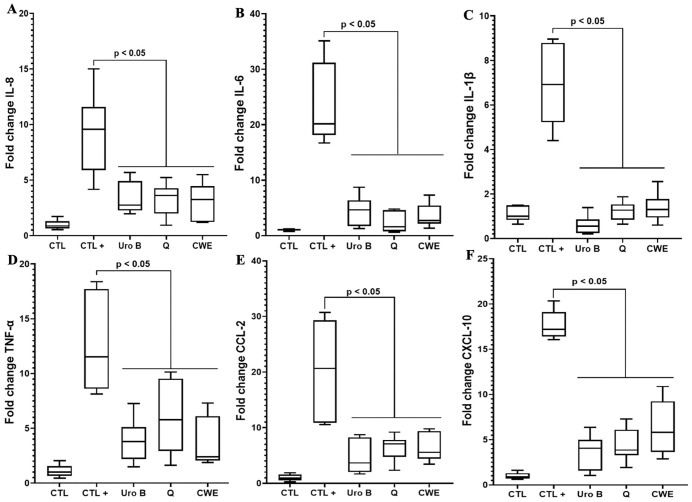
Effect of CWE treatment on the expression of pro-inflammatory mediators in human intestinal Caco-2 cells. Expression of IL-8 **(A)**, IL-6 **(B)**, IL-1β **(C)**, TNF-α **(D)**, CCL-2 (E) and CXCL-10 (F) in human intestinal Caco-2 cells challenged with 2% DSS and treated with CWE. **(A-F)**, CTL: intestinal Caco-2 cells alone; CTL + : intestinal Caco-2 cells exposed to DSS, UroB: intestinal cells exposed to DSS and treated with 100 µM UroB; Q: intestinal cells exposed to DSS and treated with 100 µM Q; CWE: intestinal cells exposed to DSS and treated with 80 µg/mL CWE. CTL indicates negative controls (untreated, non-inflamed cells), whereas CTL+ refers to positive controls representing inflammatory conditions (cells exposed to pro-inflammatory stimuli). All assays were independently repeated three times with each condition tested in triplicate per experiment (n = 9), and results are presented as mean + /- standard deviation.

In a model of intestinal Caco-2 cells challenged with DSS, we observed results consistent with those seen in LPS-stimulated macrophages. CWE reduced the expression of pro-inflammatory cytokines, including IL-6, IL-1β, and TNF-α (Fig 4). Similar to the effects of urolithin B and quercetin, CWE also decreased the expression of pro-inflammatory chemokines such as IL-8, CCL-2, and CXCL-10 (Fig 4A, 4E, and 4F). The anti-inflammatory effects of CWE were evident not only at the gene expression level but also in protein production. We quantified the levels of IL-6 and IL-1β produced by macrophages treated with CWE and exposed to LPS. CWE significantly reduced the production of these cytokines compared to controls, further supporting its anti-inflammatory properties in both intestinal Caco-2 cells and macrophages.

### The antioxidative properties of CWE in macrophages and intestinal cells

**Fig 5:**
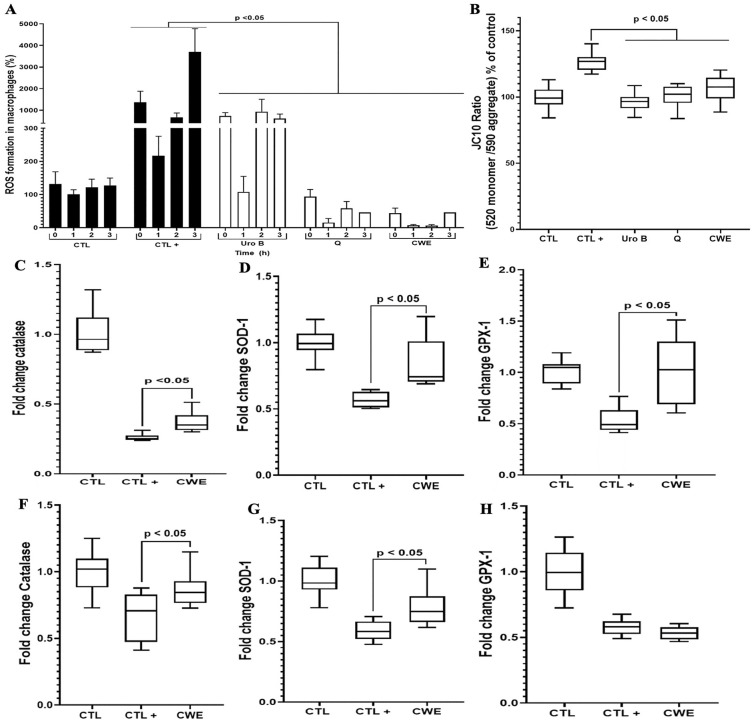
Antioxidant properties of CWE in macrophages and intestinal Caco-2 cells. Effect of CWE treatment on macrophage ROS production **(A)**, mitochondrial integrity **(B)**, and expression of catalase **(C, F)**, SOD-1 **(D, G)**, and GPX-1 (E, H) in macrophages exposed to LPS or Caco-2 cells challenged with DSS. **(A)**, CTL: macrophages alone; CTL + : macrophages exposed to LPS; UroB: macrophages exposed to LPS and treated with 100µM UroB; Q: macrophages exposed to LPS and treated with 100 µM Q; CWE: macrophages exposed to LPS and treated with 80 µg/mL CWE. **(B)**, CTL: intestinal cells alone; CTL + : intestinal cells exposed to DSS; UroB: intestinal cells exposed to DSS and treated with 100 µM of UroB; Q: intestinal cells exposed to DSS and treated with 100 µM Q; CWE: intestinal cells exposed to DSS and treated with 80 µg/mL of CWE. **(C-E)**, CTL: macrophages alone; CTL + : macrophages exposed to LPS; CWE: macrophages exposed to LPS and treated with 80 µg/mL of CWE. **(F-H)**, CTL: intestinal cells alone; CTL + : intestinal cells exposed to DSS; CWE: intestinal cells exposed to DSS and treated with 80 µg/mL of CWE. CTL indicates negative controls (untreated, non-inflamed cells), whereas CTL+ refers to positive controls representing inflammatory conditions (cells exposed to pro-inflammatory stimuli). All assays were independently repeated three times with each condition tested in triplicate per experiment (n = 9), and results are presented as mean + /- standard deviation.

In addition to their anti-inflammatory effects, polyphenols, including ellagitannins, exhibit notable antioxidant properties. These properties can be attributed to their ability to directly scavenge reactive oxygen species (ROS) or to modulate the balance between pro-oxidant and antioxidant enzymes (Fig 5). Polyphenols achieve this by enhancing the expression and production of key antioxidant enzymes, such as superoxide dismutase (SOD-1), glutathione peroxidase (GPX-1), and catalase. SOD-1, GPX-1, and catalase were selected as representative antioxidant enzymes due to their crucial roles in cellular defense against oxidative stress. Of note, we evaluated the antioxidant potential of this natural extract in macrophages exposed to LPS, providing insights into its dual role in mitigating inflammation and oxidative damage (Fig 5).

Similar to quercetin, CWE significantly reduced ROS production in macrophages. Moreover, CWE demonstrated a stronger antioxidant effect compared to urolithin B, both at the start of the experiment (T0) and up to 3 hours later (Fig 5A). These findings indicate that CWE is more effective at reducing ROS production in macrophages than either urolithin B or quercetin alone. Mitochondria play a pivotal role in ROS production through the mitochondrial respiratory chain, making them central to cellular oxidative stress (Fig 5B). To further understand CWE’s effects, we investigated its ability to mitigate ROS-induced damage to mitochondrial membrane integrity during inflammation. Consistent with the effects of established modulators like quercetin and urolithin B, CWE treatment significantly reduced the ratio of the JC-10 monomer (indicative of pro-apoptotic mitochondrial forms) to the JC-10 aggregate (indicative of healthy mitochondria). This suggests that CWE helps preserve mitochondrial membrane potential under inflammatory conditions. While CWE’s antioxidant effects on macrophages are clear, it remains uncertain whether these effects are due to direct interaction with ROS or modulation of oxidative stress-regulating enzymes. To address this, we examined the impact of CWE on the expression of antioxidative enzymes in intestinal Caco-2 cells. In macrophages exposed to inflammatory conditions, CWE treatment increased the expression of SOD-1, GPX-1, and catalase enzymes (Fig 5C, 5D, and 5E). Similarly, in human intestinal Caco-2 cells, CWE treatment enhanced the expression of SOD-1 and catalase enzymes compared to controls, although no changes in GPX-1 expression were observed (Fig 5F, 5G, and 5H). Due to prior evidence from in vitro and in vivo studies demonstrating the effects of urolithin B and quercetin on catalase, SOD-1, and GPX-1 expression under inflammatory conditions, these controls were excluded from the gene expression analyses. These results demonstrate that CWE reduces oxidative stress in both macrophages and intestinal cells by promoting the expression of antioxidant enzymes.

### The effect of CWE on the reinforcement of the intestinal barrier

**Fig 6:**
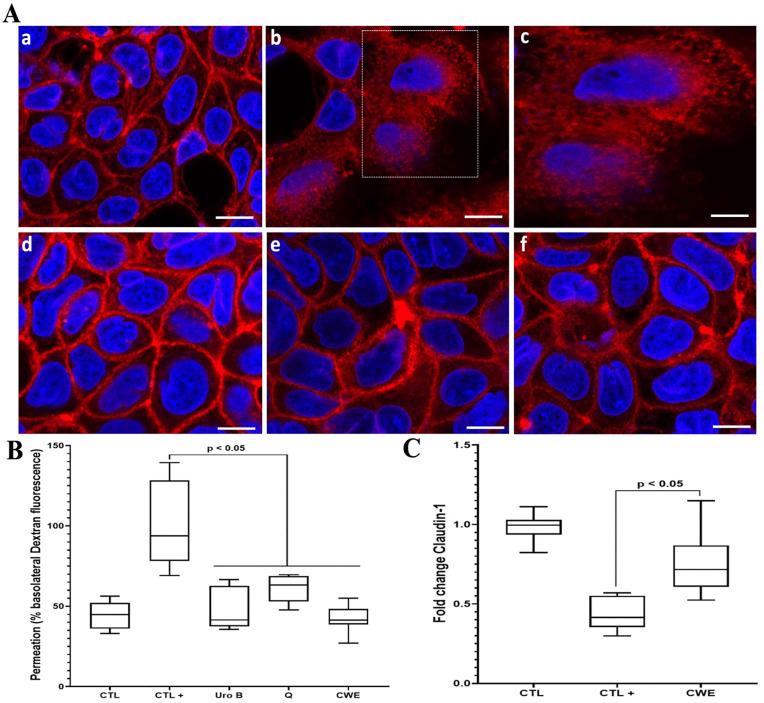
Impact of CWE treatment on intestinal barrier permeability. A, Fluorescence microscopy observation of Caco-2 cells challenged with 2%DSS and treated with CWE. Intestinal Caco-2 cells alone **(a)**; Intestinal Caco-2 cells exposed to DSS **(b, c)**; Intestinal cells exposed to DSS and treated with 100 µM of UroB **(d)**; Intestinal cells exposed to DSS and treated with 100 µM Q **(e)**, intestinal Caco-2 cells exposed to DSS and treated with 80 µg/mL of CWE **(f)**. Panel (c) is a zoomed-in view of panel **(b)**. Scale bar 5 µm for a, b, d, e and f and 2.5 µm for **c.** B, C, Intestinal permeability and tight junction expression Claudin-1 in human intestinal Caco-2 cells challenged with 2% DSS and treated with CWE. CTL: intestinal cells alone; CTL + : intestinal cells exposed to DSS, UroB: intestinal cells exposed to DSS and treated with 100 µM UroB; Q: intestinal cells exposed to DSS and treated with 100 µM Q; CWE: intestinal cells exposed to DSS and treated with 80 µg/mL CWE. CTL indicates negative controls (untreated, non-inflamed cells), whereas CTL+ refers to positive controls representing inflammatory conditions (cells exposed to pro-inflammatory stimuli). All assays were independently repeated three times with each condition tested in triplicate per experiment (n = 9), and results are presented as mean + /- standard deviation.

The data above indicate that CWE treatment reduces the inflammatory response in both macrophages and intestinal cells by decreasing the production of pro-inflammatory mediators via the PPAR-γ/NF-κB signaling pathway. To evaluate whether CWE treatment could strengthen the intestinal barrier, we utilized an *in vitro* transwell insert model with human intestinal Caco-2 cells exposed to 2% DSS and Dextran-IFTC. In this model, we measured the percentage of Dextran-IFTC passing through the Caco-2 cell barrier to assess improvements in intestinal barrier integrity. Additionally, we analyzed the effect of CWE on the expression of tight junctions in these intestinal cells, providing further insight into its potential to reinforce the intestinal barrier (Fig 6).

When the Caco-2 intestinal cell barrier is treated with urolithin B, quercetin, or CWE, the percentage of Dextran-IFTC passing through the barrier is significantly reduced compared to untreated control cells. This reduction indicates an improvement in intestinal barrier integrity (Fig 6A, and 6B). Furthermore, analyses of the tight junction protein Claudin-1 reveal that the reinforcement of tight junction proteins contributes to decreased intestinal permeability (Fig 5C). Notably, CWE treatment resulted in a significant increase in Claudin-1 expression compared to DSS controls, highlighting its potential to strengthen the intestinal barrier and reduce permeability (Fig 6A, 6B, and 6C).

### Effect of CWE on inflammatory response modulation in a *C. elegans* model infected with *C. albicans*

**Fig 7:**
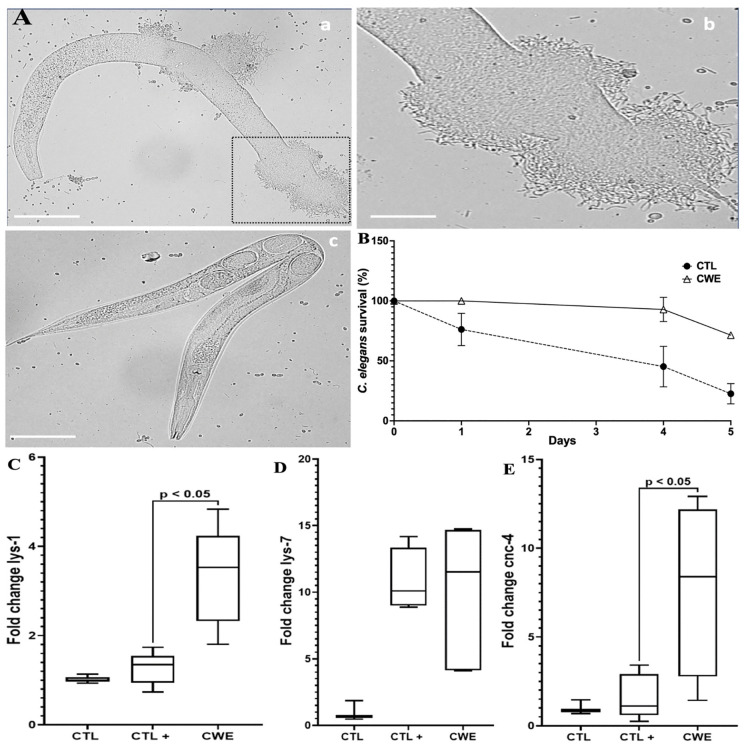
Assessing the therapeutic potential of CWE in *C. elegans* infected with *C. albicans.* A, Microscopy observation of *C. elegans* infected with *C. albicans* and treated with CWE. The nematodes were infected with *C. albicans* for 6 hours **(a,b)**. Following the infection with *C. albicans*, nematodes were treated with 100 µg/mL CWE **(c)**. Scale bar 50 µm for a, and c and 25 µm for **c.** B, During the course of the experiment, nematodes infected with *C. albicans* and treated with CWE were quantified to determine their survival rate over time. CTL corresponds to nematodes infected with *C. albicans* and untreated with CWE; CWE corresponds to nematodes infected with *C. albicans* and treated with 100 µg/mL of CWE. C, D, and E, Expression of Lys-1, Lys-7 and Cnc-4 in *C. elegans* infected with *C. albicans* and treated with CWE. CTL corresponds to nematodes infected with *C. albicans* and untreated with CWE; CWE corresponds to nematodes infected with *C. albicans* and treated with 100 µg/mL of CWE.

After analyzing CWE’s effects on inflammatory responses in intestinal cells and macrophages, we employed a nematode model infected with *C. albicans* to investigate its effects *in vivo*. The *C. elegans* model allows for a more complex analysis of the inflammatory process compared to cellular models. In this setup, *C. elegans* were infected with *C. albicans* for 6 hours before being treated with 100 µg/mL of CWE. Subsequently, we assessed the survival of CWE-treated nematodes against *C. albicans* infection over time, providing valuable insights into the *in vivo* efficacy of CWE in mitigating infection-related inflammation (Fig 7).

The survival rate of nematodes treated with CWE was significantly higher compared to untreated nematodes (control group) when exposed to *C. albicans* (Fig 7A and 7B). We further analyzed the immune response of *C. elegans* infected with *C. albicans* and treated with CWE. In nematodes infected with *C. albicans*, CWE treatment boosted immune defenses by increasing the expression of Cnc-4 and Lys-1, a key nematode enzyme involved in immune defense against pathogens (Fig 7C, 7D, and 7E). These findings align with previous analyses of CWE’s anti-inflammatory effects in cellular models and its protective role against pathogen infection, reinforcing the extract’s potential as an immunomodulatory agent.

## Discussion

Therapeutic challenges in IBD, due to treatment limitations and side effects, highlight the need for new approaches [7,8]. Natural compounds like resveratrol, quercetin, and curcumin show promise by simultaneously modulating inflammation and oxidative stress, offering a holistic strategy to restore intestinal homeostasis and support mucosal health [24,25]. Polyphenols exhibit broad therapeutic potential, acting synergistically with chemotherapeutics in cancer and providing protective, immunomodulatory effects in chronic inflammatory diseases [37,38]. In IBD, they support gut barrier integrity, enhance microbiota diversity, and reduce tissue damage, highlighting their promise as multifunctional agents for both oncology and inflammation management [37,39].

Polyphenols are also known for their varied modes of action, resulting in a variety of secondary metabolites that are beneficial to human health by interfacing with their host and intestinal microbiota [38]. It is important to acknowledge that polyphenols exhibit limited intestinal absorption and undergo extensive biotransformation. In addition, high concentrations of certain polyphenols may exert pro-oxidant or cytotoxic effects depending on the cellular context [14,15].

In the present study, we screened various natural extracts of plant origin (light oak wood extract, dark oak wood extract, cherry wood extract, cashew extract, etc). CWE contains ellagitannin, a water-soluble tannin whereas other wood extracts used in the present study are non-hydrolyzable tannin scaffolds composed of catechins and anthocyanidin aglycones [31]. We found that CWE reduced NF-κB expression in LPS-stimulated macrophages compared to other natural products. In light of these data, we selected CWE as the extract of choice for the following experiments. Building on this plant extract selection, the macrophage assays conducted in this study were designed to explore underlying mechanistic pathways. In terms of CWE, the composition of the native extract used in vitro does not necessarily reflect the molecular forms that ultimately reach macrophages in vivo following oral administration. Growing evidence indicates that ellagitannins and ellagic acid are extensively metabolized by the gut microbiota into urolithins, smaller and more readily absorbed metabolites that circulate predominantly as glucuronide and sulfate conjugates [40]. In different studies using pomegranate or ellagitannin-rich sources, total circulating urolithin concentrations have been reported to reach up to 18 µM in certain individuals, although with marked interindividual variability [41]. These data support the biological relevance of micromolar exposure levels for polyphenol-derived metabolites. The concentration of 80 μg/mL used in the present study refers to the crude extract and does not correspond to 80 μg/mL of a single purified bioactive compound. Quantitative profiling indicated that hydrolyzable tannins represent approximately 76.7% of total polyphenols, while individual constituents are present at substantially lower concentrations [42]. Thus, the effective concentration of each bioactive molecule is considerably lower than the nominal extract concentration. Moreover, this dose was experimentally validated as non-cytotoxic up to 300 μg/mL and remains within the range commonly used in mechanistic in vitro studies investigating the cellular and molecular effects of dietary polyphenols.

Our findings suggest that CWE exerts anti-inflammatory effects by reducing key pro-inflammatory cytokines and chemokines in macrophages and intestinal cells under inflammatory conditions, highlighting its potential role in modulating immune responses. These results align with findings from Pozzoli et al., who reported that CWE inhibited the release of pro-inflammatory mediators (CXCL-10, IL-8, MCP-1, ICAM) in Caco-2 cells treated with IL-1β and INF-γ cocktails [29].

Moreover, CWEs have been widely utilized as natural alternatives to antibiotics in veterinary medicine for managing infectious pathologies in livestock, including cattle, pigs, and poultry [13]. CWE treatment enhances the expression of PPAR-γ, which plays a critical role in reducing the production of inflammatory mediators by modulating the NF-κB signaling pathway [25]. The involvement of PPARγ in the anti-inflammatory activity of the extract is strongly supported by both our experimental data and previously published studies [43–45]. Of note, multiple reports have demonstrated that polyphenolic compounds such as ellagitanins or urolithins (metabolites derived from chestnut wall polyphenols) can activate PPARγ and inhibit NF-κB signaling, thereby exerting anti-inflammatory effects [43–45]. Consistent with our findings, CWE treatment significantly reduced NF-κB activity in THP-1-Lucia™ cells, supporting a mechanism involving PPARγ activation and subsequent NF-κB inhibition. Of note, our findings suggest that CWE modulates macrophage-mediated inflammation through both PPARγ-dependent and -independent mechanisms. The differential regulation of IL-1β and IL-6, particularly the persistence of IL-6 reduction despite PPARγ inhibition, highlights the involvement of additional signaling pathways beyond PPARγ activation.

Additionally, we observed that CWE treatment reduces ROS production in macrophages and protects mitochondrial membrane integrity against ROS-induced damage. This protective effect is closely associated with increased PPAR-γ expression. Consistent with our findings, Wu et al. demonstrated that quercetin treatment improved mitochondrial membrane potential, facilitated the removal of damaged mitochondria, and decreased ROS accumulation, leading to a significant reduction in both cell apoptosis and inflammatory responses [46]. These combined effects highlight the potential of CWE as a therapeutic agent for mitigating inflammation and oxidative stress.

We showed that CWE treatment enhances the expression of antioxidant enzymes, including SOD-1, GPX-1, and catalase, in both macrophages and intestinal epithelial Caco-2 cells. Our findings align with previous studies that have consistently reported the antioxidant effects of CWE, including increased expression and activity of key antioxidant enzymes [47–49]. These established observations strongly support the antioxidant role of CWE in our model [47–49]. Liu et al. reported that broilers fed a CWE-enriched diet exhibited higher total antioxidant capacity, GPX-1, and SOD-1 values in breast and thigh muscles compared to those fed a diet containing antibiotics [50]. In addition to its antioxidant effects, CWE treatment reinforced the intestinal barrier by improving the expression of tight junction proteins and mitigating the detrimental effects of DSS on Caco-2 cell barriers. These findings are consistent with data showing increased occludens-1 mRNA levels in broilers fed a CWE-supplemented diet [50]. Furthermore, in a clinical setting, CWE treatment demonstrated additional benefits by improving microbiota biodiversity and reducing intestinal permeability in COVID-19 patients [10]. These findings underscore the multifaceted therapeutic potential of CWE in enhancing antioxidant defenses, preserving gut barrier integrity, and promoting microbiota health in both preclinical and clinical contexts. CWE significantly enhances the survival of *C. elegans* exposed to C. albicans suggesting a protective effect against fungal infection. CWE treatment led to the upregulation of Cnc-4, a gene encoding a cecropin-like antimicrobial peptide involved in pathogen clearance, as well as Lys-1 and Lys-7, which encode lysozymes critical for degrading bacterial and fungal cell walls. The increased expression of these immune effectors indicates an activation of the nematode’s innate immune response [35,51]. This immunostimulatory activity is consistent with our findings from cellular models demonstrating the anti-inflammatory properties of CWE.

It is noteworthy that the beneficial effects of the CWE used in this study are already well documented in livestock [47,52,53]. This extract is commercially available in France and is widely incorporated into animal feed, particularly in pasture-based systems, to reduce antibiotic use and enhance immune defenses against infections. The anti-inflammatory properties observed in animals are in line with the effects demonstrated in the present in vitro cell-based assays and *C. elegans* model, supporting the broader relevance of these findings. However, in vitro models, while offering controlled and reproducible experimental conditions, cannot fully capture the complexity of multicellular interactions and systemic immune responses. Similarly, *C. elegans*, although a valuable whole-organism model, lacks an adaptive immune system and some features of mammalian physiology. To address these limitations and further substantiate the current results, ongoing studies using a DSS-induced colitis mouse model are underway, which will help confirm and extend these observations in a more physiologically relevant setting. Besides, identifying the specific bioactive constituents underlying the anti-inflammatory effects of CWE is a key objective moving forward. Collaborative efforts with chemists are underway to assess the biological activity of individual compounds isolated from the extract.

In conclusion, CWE shows significant anti-inflammatory and antioxidant properties, making it a promising therapeutic agent for managing conditions associated with inflammation and oxidative stress. CWE modulates the inflammatory response by reducing the expression of key pro-inflammatory mediators, including IL-6, IL-1β, and TNF-α. Additionally, it enhances the activity of antioxidant enzymes such as SOD-1 and GPX-1, protecting cells from damage caused by free radicals. CWE also promotes the expression of PPAR-γ, which contributes to a balanced immune response by improving mitochondrial membrane potential and reducing oxidative damage. These effects collectively provide a protective mechanism against cellular stress linked to inflammation. Furthermore, CWE treatment strengthens the intestinal barrier by enhancing the expression of tight junctions and mitigating the detrimental effects of DSS on Caco-2 cell barriers. *In vivo* studies further highlight its protective effects, such as its ability to enhance the host’s immune response against *C. albicans* infection in *C. elegans*. These combined properties underscore the potential therapeutic applications of CWE in addressing inflammatory and oxidative stress-related conditions, offering a natural and effective approach to improving health outcomes.

## Supporting information

S1 Table Polyphenol Characterization of CWE by UPLC-DAD-MS. (DOCX)

S1 Fig Identification and characterization of the polyphenolic composition of CWE using UHPLC-DAD analysis. (DOCX)

S2 Fig Cytotoxicity effect of CWE on macrophages and intestinal Caco-2 cells. (DOCX)

S3 Fig CWE treatment modulates inflammatory signaling pathways in DSS-challenged human intestinal Caco-2 cells. (DOCX)

S1 Data Raw data. (XLSX)
