# Supplementary material for: Turning off inflammation naturally via dual antioxidant and anti-inflammatory actions of chestnut wood extract through PPARγ and NF-κB pathways
Source: PLoS One. 2026 Apr 29;21(4):e0347987. doi: 10.1371/journal.pone.0347987 (PMC13127955; doi:10.1371/journal.pone.0347987)
Supplement: S1 Table — (DOCX) [file pone.0347987.s001.docx]

| **Time** | **% Water (A)** | **% ACN (B)** |
| --- | --- | --- |
| 0.0–0.4 | 99 | 1 |
| 0.4–5.0 | 99 → 90 | 1 → 10 |
| 5.0–9.0 | 90 → 87 | 10 → 13 |
| 9.0–11.0 | 87 → 80 | 13 → 20 |
| 11.0–13.0 | 80 → 65 | 20 → 35 |
| 13.0–14.0 | 65 → 50 | 35 → 50 |
| 14.0–15.8 | 50 → 30 | 50 → 70 |
| 15.8–17.8 | 30 → 8 | 70 → 92 |
| 17.8–18.0 | 8 → 0 | 92 → 100 |
| 18.0–19.0 | 0 | 100 |
| 19.0–19.2 | 0 → 99 | 100 → 1 |
| 0,0–0.4 | 99 | 1 |
| 0.4–5.0 | 99 → 90 | 1 → 10 |

**Table S1. Polyphenol Characterization of CWE by UPLC-DAD-MS**

CWE is a polyphenol-rich extract, particularly abundant in ellagitannins. The identification of multiple polyphenolic compounds in CWE was carried out using the chromatographic method described by Metoyer et al. [1]. This analytical protocol, based on ultra-performance liquid chromatography (UPLC) coupled with diode array detection (DAD) and electrospray ionization ion trap mass spectrometry (ESI-IT-MS), allowed the separation and structural characterization of hydrophilic ellagitannins, including both primary and secondary metabolites. Using this approach, a total of 23 distinct polyphenols were identified in CWE. Notable among these were major ellagitannins such as castalin, castalagin, vescalagin, roburins (Fig.1 C–G), and castacrenins (Fig.1 H, I, K and L), highlighting the complexity and richness of the extract’s polyphenolic profile. For analytical method based on metoyer et al [1].

The analytical system included an Agilent UPLC platform (G4226A, 1290 Sampler) controlled by HyStar acquisition software and Esquire MS Control. Detection was performed using a diode array detector (4212B, 1260 DAD) monitoring absorbance from 200 to 600 nm, and a Bruker Esquire 6000 ion trap mass spectrometer, equipped with electrospray ionization (ESI) operated in negative mode. Source parameters (Capillary: +3400 V, End plate offset: −500 V, Nebulizer: 40 psi, Dry gas flow: 10 L/min, and Dry temperature: 365 °C). For ion trap settings (Skimmer: −40 V, Capillary exit: −115.3 V, Oct 1 DC: −12 V, Oct 2 DC: −1.7 V, Trap drive: 50.7, Oct RF: 157.5 Vpp, Lens 1: 5.0 V, Lens 2: 60.0 V). For chromatographic conditions: Column: Agilent SB-C18 RRHD (2.1 × 100 mm, 1.8 μm), Guard column: Agilent SB-C18 (2.1 × 5 mm, 1.8 μm), Flow rate: 0.4 mL/min, and Injection volume: 1 µL. Mobile phase:gradient elution was performed with water (A) and acetonitrile (B), both acidified with 0.1% formic acid, as follows.

For sample preparation and quantification, CWE samples were initially dissolved in DMSO, then diluted in ultrapure water to ensure compatibility with the UPLC-DAD-MS analysis system. The Folin–Ciocalteu method was used to quantify total polyphenol content, with a specific focus on ellagitannins. For isolation and structural characterization, to isolate individual polyphenols and obtain their corresponding UV-visible and mass spectra, two fractionation techniques were employed: Centrifugal Partition Chromatography (CPC) and Size Exclusion Chromatography (SEC). Each isolated compound was characterized based on its molar mass and fragmentation ion structure, allowing full identification and confirmation of the 23 polyphenolic compounds present in CWE.

**Reference**

1. Metoyer, B.; Renouf, E.; Jourdes, M.; Merillon, J. M.; Teguo, P. W., Isolation of Hydrolyzable Tannins from Castanea sativa Using Centrifugal Partition Chromatography. *J Nat Prod* **2024,** 87, (4), 652-663.
