# Supplementary material for: Turning off inflammation naturally via dual antioxidant and anti-inflammatory actions of chestnut wood extract through PPARγ and NF-κB pathways
Source: PLoS One. 2026 Apr 29;21(4):e0347987. doi: 10.1371/journal.pone.0347987 (PMC13127955; doi:10.1371/journal.pone.0347987)
Supplement: S1 Fig — (DOCX) [file pone.0347987.s002.docx]

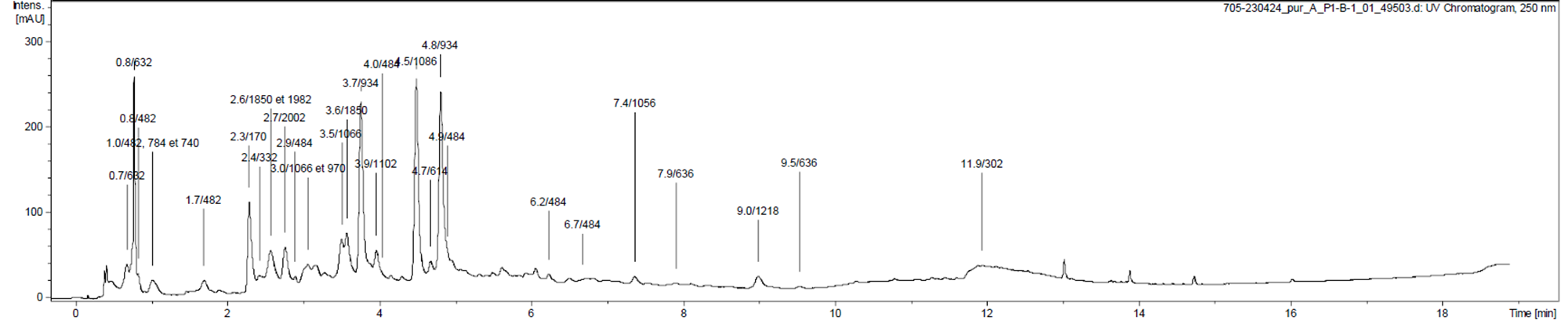


A


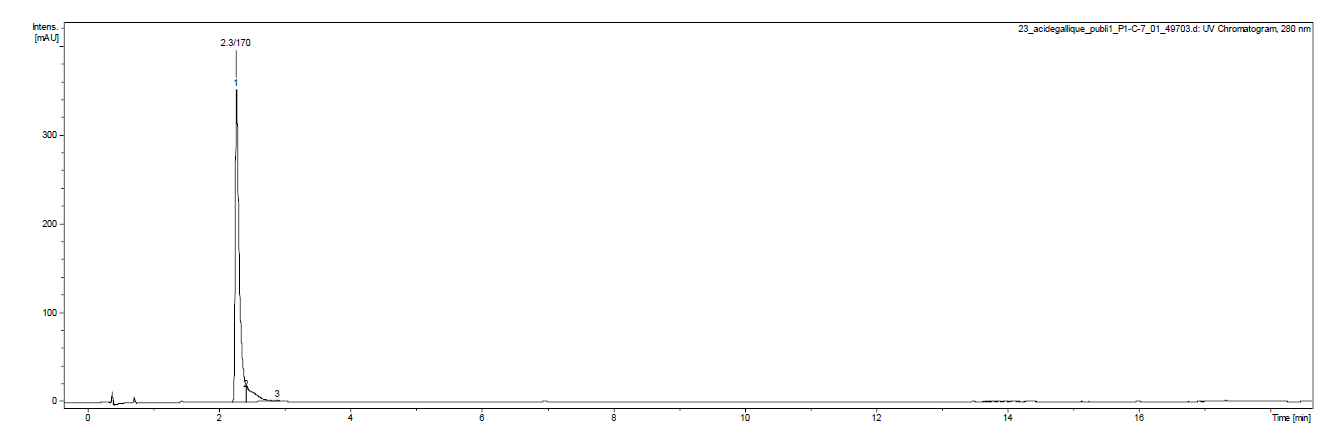


B


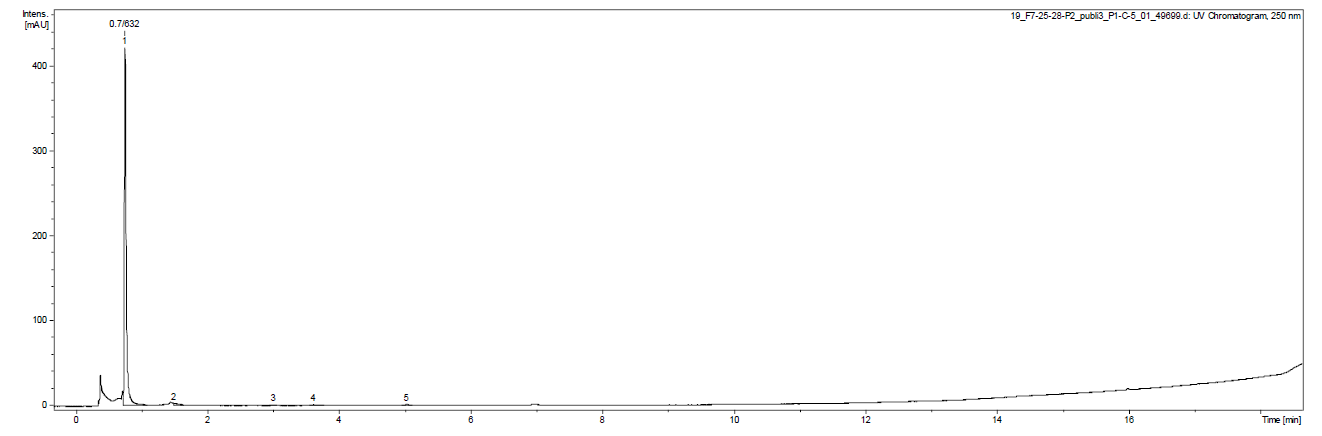


C


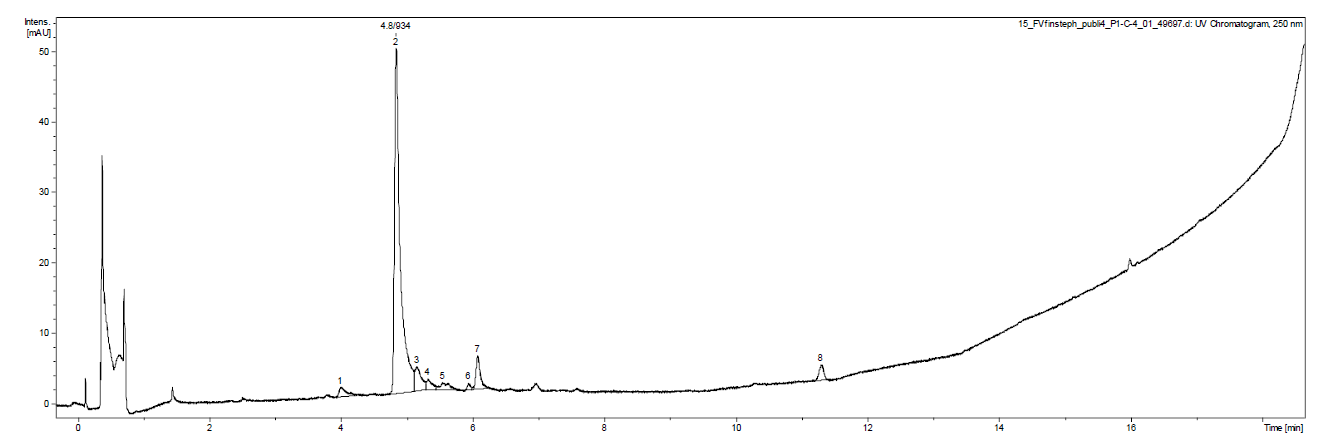


D


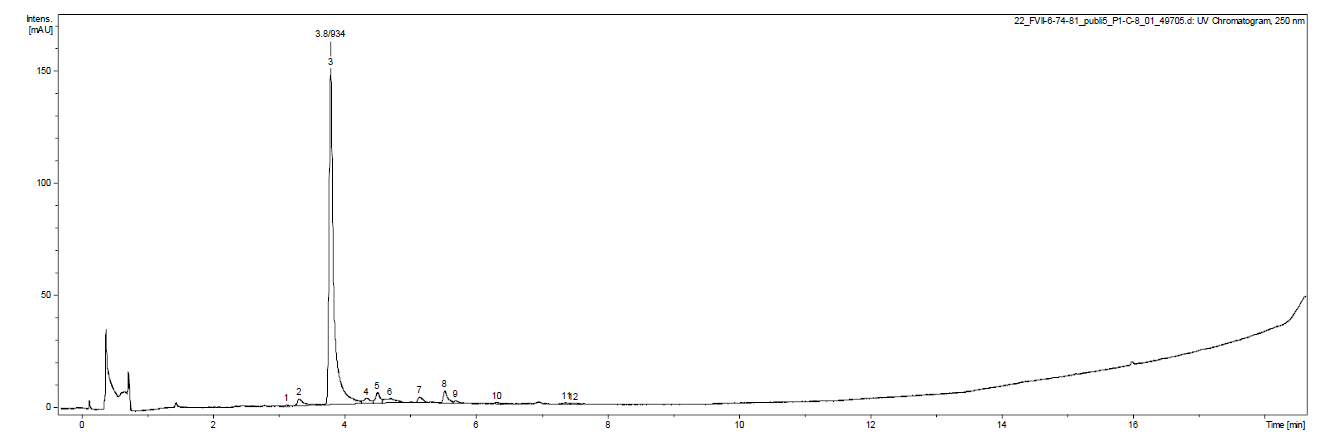


E


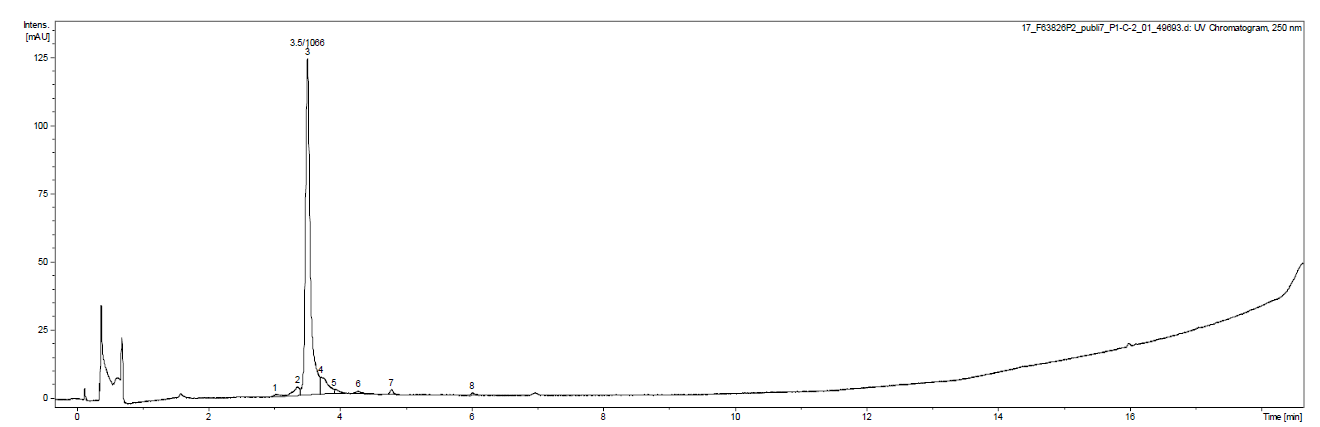


F


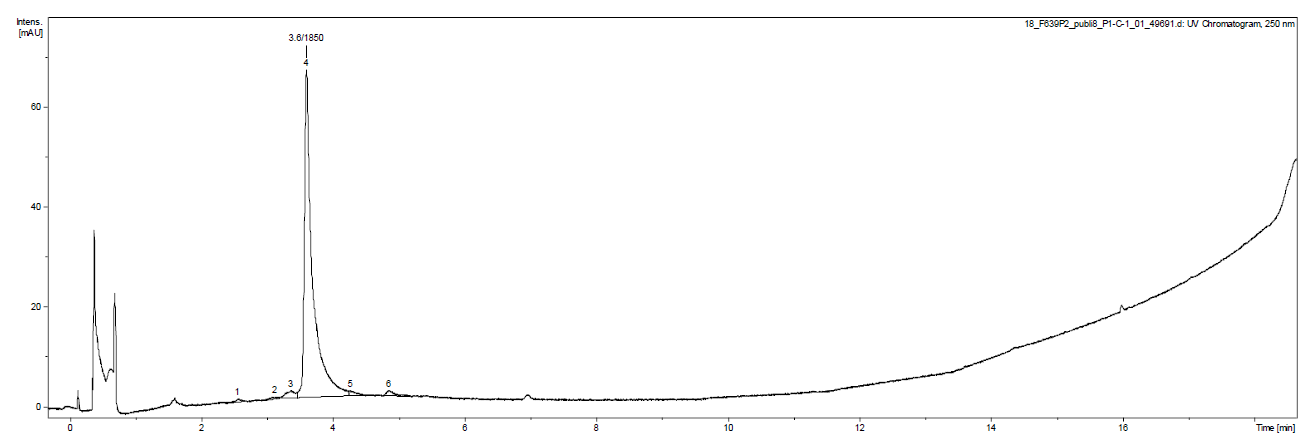


G


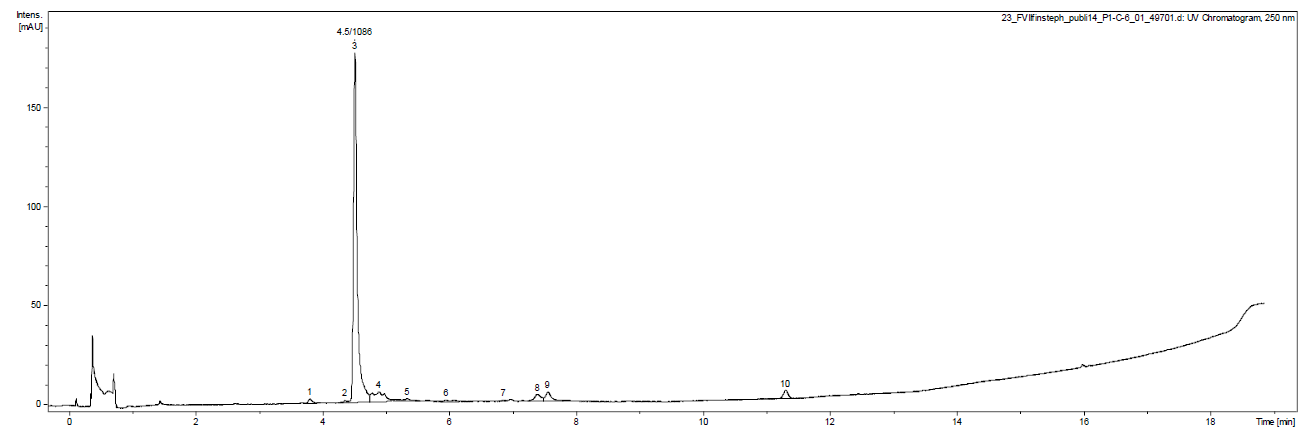


H


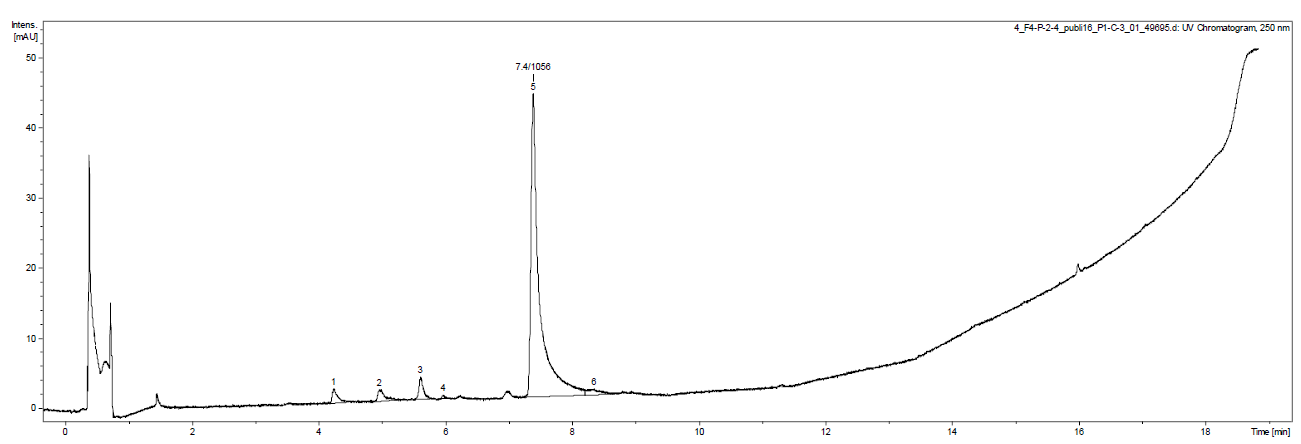


I


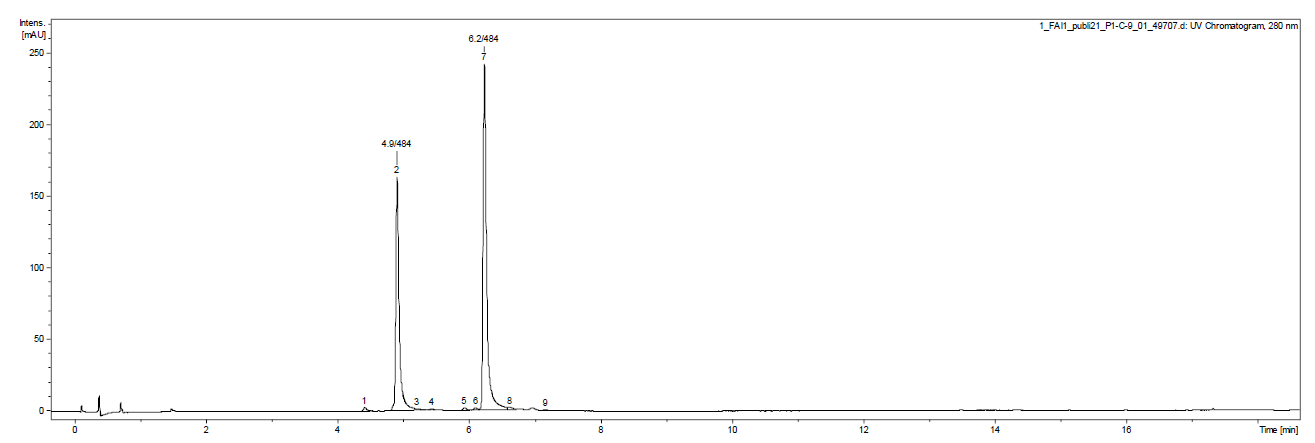


J


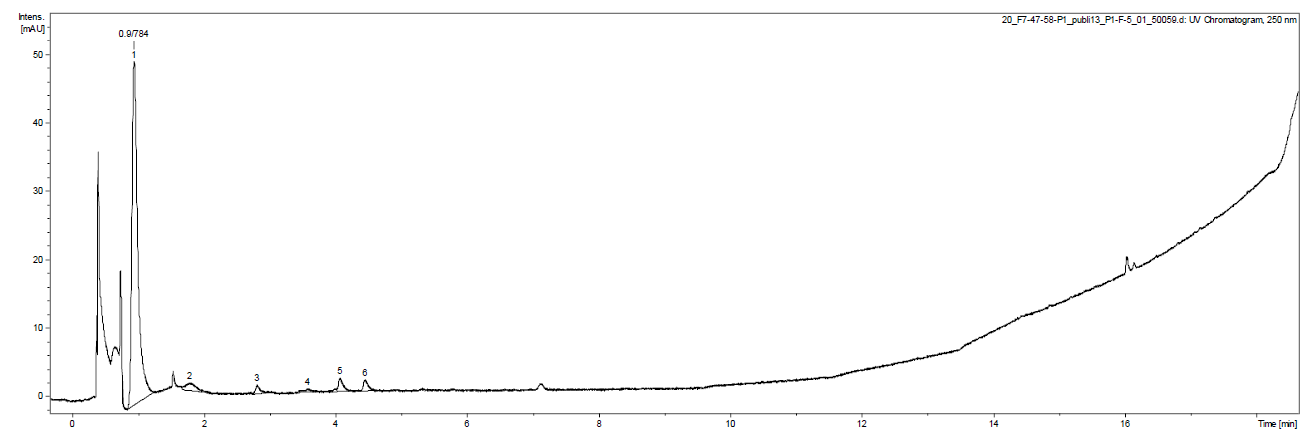


K


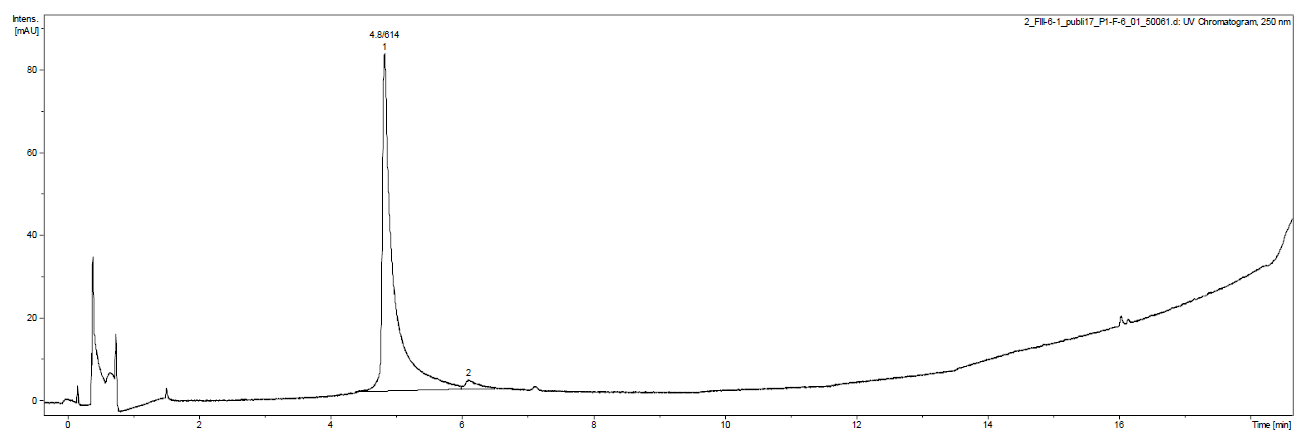


L

**Fig. S1: Identification and characterization of the polyphenolic composition of CWE using UHPLC-DAD analysis.** The UHPLC-DAD chromatograms depict the detailed polyphenol profile of the CWE sample, recorded at 250 nm. Panel (A) shows the overall chromatographic fingerprint of CWE polyphenols. Panels (B) through (L) present chromatograms of individually identified compounds within the extract: (B) gallic acid, (C) castalin, (D) castalagin, (E) vescalagin, (F) roburin E, (G) roburin D, (H) castacrenin D, (I) castacrenin E, (J) 2,6-digalloyl-α-D-glucose and 2,6-digalloyl-β-D-glucose, (K) castacrenin I, and (L) castacrenin C. These chromatograms confirm the presence and retention times of each polyphenolic compound, providing a comprehensive profile of CWE’s bioactive constituents.
