# Supplementary material for: Turning off inflammation naturally via dual antioxidant and anti-inflammatory actions of chestnut wood extract through PPARγ and NF-κB pathways
Source: PLoS One. 2026 Apr 29;21(4):e0347987. doi: 10.1371/journal.pone.0347987 (PMC13127955; doi:10.1371/journal.pone.0347987)
Supplement: S2 Fig — (DOCX) [file pone.0347987.s003.docx]

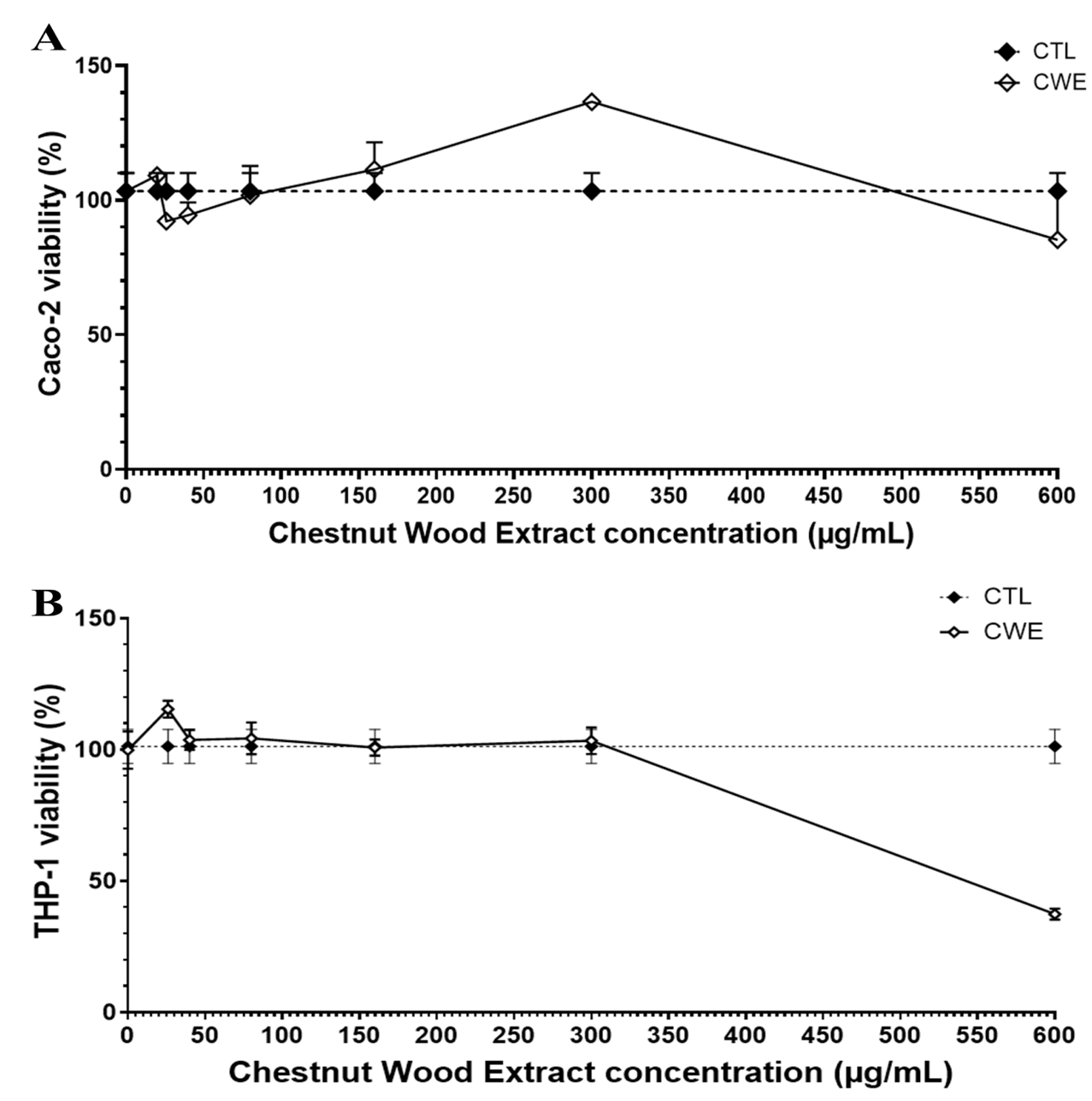


**Fig. S2: Cytotoxicity effect of CWE on macrophages and intestinal Caco-2 cells.** (A) Viability of THP-1-derived macrophages treated with increasing concentrations of CWE (20, 26, 40, 80, 160, 300, and 600 µg/mL) compared to untreated control cells (CTL). (B) Viability of human intestinal Caco-2 cells exposed to the same range of CWE concentrations relative to untreated controls (CTL). All assays were independently repeated three times with each condition tested in triplicate per experiment (n = 9), and results are presented as mean +/- standard deviation.

**Cytotoxicity Assessment of CWE on Human Macrophages and Intestinal Epithelial Cells**

To ensure that the observed anti-inflammatory and antioxidant effects of CWE were not related to cytotoxicity-induced cell damage, we carried out comprehensive cytotoxicity assays on both THP-1-derived macrophages and human intestinal epithelial Caco-2 cells. These experiments were specifically designed to exclude the possibility that reductions in inflammatory markers and oxidative stress were artifacts of compromised cell viability (Fig. S2).

Cytotoxicity was assessed across a range of CWE concentrations, with particular attention to doses exceeding those employed in the functional assays. Of note, no cytotoxic effects were detected in either cell type at concentrations up to 300 µg/mL, four times higher than the maximum concentration used in the mechanistic experiments. Cell morphology, viability, and density remained comparable to untreated controls across all tested conditions. These findings strongly indicate that the biological activities attributed to CWE, including its capacity to modulate inflammatory and oxidative responses, are not due to non-specific cytotoxic effects.

**Cytotoxicity Assay on Intestinal and Macrophage Cells**

Caco-2 and THP-1 cells were Incubated at a density of 1 × 10^5^ cells/mL in 96-well plates. Cells were then treated with or without CWE at concentrations of 20, 26, 40, 80, 160, 300, and 600 µg/mL and incubated for 24 hours at 37° C in a humidified atmosphere containing 5% CO₂. Following incubation, 10 µL of MTT reagent (tetrazolium salt; Catalog No. 4890-25-01; Biotechne / R&D Systems, Germany) was added to each well, and plates were further incubated for 3 hours under the same conditions. Viable cells reduce the yellow tetrazolium salt to insoluble blue formazan crystals. Subsequently, a detergent reagent (Catalog No. 4890-25-01; Biotechne / R&D Systems, Germany) was added to dissolve the formazan crystals, and cell viability was quantified by measuring the optical density at 570 nm using a spectrophotometer.
