# Supplementary material for: Turning off inflammation naturally via dual antioxidant and anti-inflammatory actions of chestnut wood extract through PPARγ and NF-κB pathways
Source: PLoS One. 2026 Apr 29;21(4):e0347987. doi: 10.1371/journal.pone.0347987 (PMC13127955; doi:10.1371/journal.pone.0347987)
Supplement: S3 Fig — (DOCX) [file pone.0347987.s004.docx]

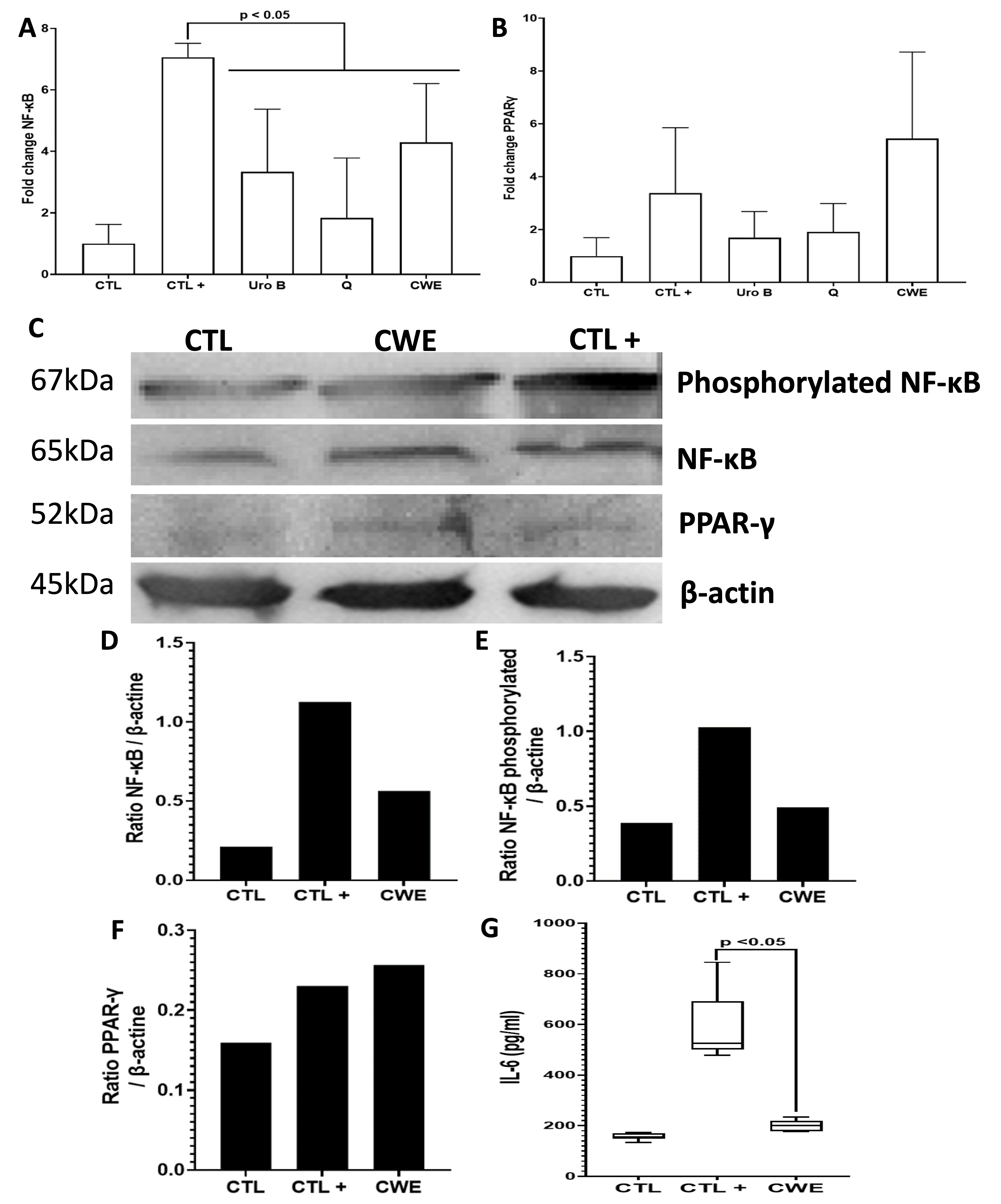


**Fig. S3: CWE treatment modulates inflammatory signaling pathways in DSS-challenged human intestinal Caco-2 cells.** The effects of CWE on inflammatory mediator expression were evaluated in Caco-2 cells exposed to 2% DSS. (A and B) Relative mRNA expression of NF-κB and PPAR-γ. (C-F) Representative immunoblots and densitometric quantitative analysis of phosphorylated NF-κB, total NF-κB, and PPAR-γ protein expression. (E-F) Densitometric analysis of band intensity was performed using ImageJ. CTL: untreated, non-inflamed cells (negative control); CTL+: DSS-exposed cells (inflammatory positive control); CWE: DSS-exposed cells treated with 80 µg/mL CWE. Protein expression levels were normalized to β-actin and expressed as relative ratios. (G) Effect of CWE treatment on pro-inflammatory protein expression in human intestinal Caco-2 cells. IL-6 protein production was quantified by Elisa in Caco-2 cells exposed to DSS and subsequently treated with CWE. CTL: untreated Caco-2 cells; CTL+: Caco-2 cells exposed to DSS; CWE: Caco-2 cells exposed to DSS and treated with 80 µg/mL CWE. All experiments were independently performed twice with each condition tested in triplicate (n = 6). Data are presented as mean ± standard deviation.
